# MAP3K4 promotes fetal and placental growth by controlling the receptor tyrosine kinases IGF1R/IR and Akt signaling pathway

**DOI:** 10.1016/j.jbc.2022.102310

**Published:** 2022-07-31

**Authors:** Charles H. Perry, Nathan A. Mullins, Razan B.A. Sweileh, Noha A.M. Shendy, Patrick A. Roberto, Amber L. Broadhurst, Hannah A. Nelson, Gustavo A. Miranda-Carboni, Amy N. Abell

**Affiliations:** 1 Department of Biological Sciences, University of Memphis, Memphis, Tennessee, USA; 2 Division of Molecular Oncology, Department of Oncology, St. Jude Children's Research Hospital, Memphis, Tennessee, USA; 3 University of Tennessee Health Science Center, Department of Medicine, Division of Hematology-Oncology, Center for Cancer Research, Memphis, Tennessee, USA

**Keywords:** MAP3K4, protein kinase, fetal growth restriction, Akt/PKB, insulin-like growth factor receptor, insulin receptor, placenta, insulin-like growth factor, Akt, protein kinase B, CBP, CREB-binding protein, CDX2, caudal homeobox 2, DMSO, dimethyl sulfoxide, E-cadherin, epithelial cadherin, EMT, epithelial-to-mesenchymal transition, ERBB2, Erb-B2 receptor tyrosine kinase 2, ERK2, extracellular-regulated kinase 2, FGF4, fibroblast growth factor 4, FGFR4, fibroblast growth factor receptor 4, FGR, fetal growth restriction, GALNT3, O-GalNAc glycosyltransferase 3, GSK3α, glycogen synthase kinase 3 alpha, GSK3β, glycogen synthase kinase 3 beta, HDAC6, histone deacetylase 6, IGF, insulin-like growth factor, IGF1R, insulin-like growth factor 1 receptor, IR, insulin receptor, JNK, c-Jun N-terminal kinase, KI, kinase inactivation, MAPK, mitogen-activated protein kinase, MAP3K4, mitogen-activated protein kinase kinase kinase 4, MEF-CM, mouse embryonic fibroblast conditioned media, PDK1, 3-phosphoinositide-dependent protein kinase 1, PTEN, phosphatase and tensin homolog, qPCR, quantitative PCR, RTK, receptor tyrosine kinase, Ser473, serine 473, SynT-II, syncytiotrophoblast layer II, TE, trophectoderm, Thr308, threonine 308, TS, trophoblast stem

## Abstract

Disruption of fetal growth results in severe consequences to human health, including increased fetal and neonatal morbidity and mortality, as well as potential lifelong health problems. Molecular mechanisms promoting fetal growth represent potential therapeutic strategies to treat and/or prevent fetal growth restriction (FGR). Here, we identify a previously unknown role for the mitogen-activated protein kinase kinase kinase 4 (MAP3K4) in promoting fetal and placental growth. We demonstrate that inactivation of MAP3K4 kinase activity causes FGR due in part to placental insufficiency. Significantly, MAP3K4 kinase–inactive mice display highly penetrant lethality prior to weaning and persistent growth reduction of surviving adults. Additionally, we elucidate molecular mechanisms by which MAP3K4 promotes growth through control of the insulin-like growth factor 1 receptor (IGF1R), insulin receptor (IR), and Akt signaling pathway. Specifically, MAP3K4 kinase inactivation in trophoblast stem (TS) cells results in reduced IGF1R and IR expression and decreased Akt activation. We observe these changes in TS cells also occur in differentiated trophoblasts created through *in vitro* differentiation of cultured TS cells and *in vivo* in placental tissues formed by TS cells. Furthermore, we show that MAP3K4 controls this pathway by promoting *Igf1r* transcript expression in TS cells through activation of CREB-binding protein (CBP). In the MAP3K4 kinase–inactive TS cells, *Igf1r* transcripts are repressed because of reduced CBP activity and increased histone deacetylase 6 expression and activity. Together, these data demonstrate a critical role for MAP3K4 in promoting fetal and placental growth by controlling the activity of the IGF1R/IR and Akt signaling pathway.

Fetal growth and development are absolutely dependent on the placenta to efficiently deliver nutrients and oxygen to the developing fetus. Glucose, essential amino acids, and hormones necessary for proper fetal growth and development are actively transported from the placenta to the growing fetus (1). Placental insufficiency results in the failure to reach an individual’s genetically predetermined size, defined as fetal growth restriction (FGR) or intrauterine growth restriction (2, 3). FGR affects up to 10% of human pregnancies and is associated with increased rates of miscarriage, preterm birth, morbidity, and mortality of the fetus (2, 4, 5, 6). The standard for FGR identification is transvaginal ultrasound, although this method has been associated with misdiagnosis (7). Long-term consequences of FGR include increased fetal susceptibility to infection, neonatal neurodevelopmental impairments, and development of metabolic disorders later in life (8, 9, 10). Importantly, placental insufficiency is the leading cause of FGR, highlighting the critical role of normal placental function to the developing embryo and fetus (11). Thus, the identification of placental enzymes preventing FGR may offer insight into early therapeutic strategies.

The placenta is formed from trophoblast subtypes derived from the trophectoderm (TE), endothelial cells derived from the fetal mesenchyme, and decidual cells derived from the mother (12). TE-derived cells undergo extensive cell proliferation and differentiation to form the trophoblasts of the placenta. A functional placenta is absolutely required from E10 onward in the mouse. After E12.5, placental growth occurs primarily through trophoblast cell hypertrophy instead of cellular proliferation (13). Trophoblast stem (TS) cells isolated at E3.5 from the TE of WT preimplantation blastocysts (TS^WT^) can be cultured indefinitely in the presence of fibroblast growth factor 4 (FGF4) and mouse embryonic fibroblast conditioned media (MEF-CM) (14). Removal of these factors induces differentiation to all trophoblast subtypes, including syncytiotrophoblasts, spongiotrophoblasts, and trophoblast giant cells (14, 15). Mitogen-activated protein kinase kinase kinase 4 (MAP3K4) is expressed highly in TS cells and tissues derived from TS cell differentiation (15). In response to mainly stress stimuli like heat shock, MAP3K4 phosphorylates the mitogen-activated protein kinase kinases MAP2K4/7 and MAP2K3/6, which phosphorylate and activate the downstream mitogen-activated protein kinases (MAPKs) c-Jun N-terminal kinase (JNK) and p38, respectively (15, 16). A targeted point mutation of the endogenous MAP3K4 active-site lysine at position 1361 with an arginine results in kinase inactivation (KI) of MAP3K4 (*Map3k4*^*KI/KI*^) (16). TS cells with MAP3K4 KI (TS^KI^) maintain stemness but display reduced FGF4-stimulated phosphorylation of JNK and p38 (15, 17). MAP3K4 KI results in early developmental defects of the placenta including but not limited to implantation defects and defective decidualization (15). Furthermore, MAP3K4 KI embryos in a mixed 129/SvEv/C57BL/6N background display developmental defects, including exencephaly, spina bifida, and male gonadal sex reversal demonstrating that MAP3K4 is critical for normal development (16, 18). However, the role of MAP3K4 in placental and fetal growth remains poorly understood.

Fetal development and growth are dependent on multiple cellular processes, including proliferation, differentiation, and apoptosis (19). These processes require tight hormonal regulation that occurs in fetal gestation and continues in postnatal life. Insulin-like growth factor 1 receptor (IGF1R) and the insulin receptor (IR) are closely related receptor tyrosine kinases that form homodimers and heterodimers with one another, recognizing the ligands insulin-like growth factor 1 (IGF1), IGF2, and insulin (20). Upon ligand binding, IGF1R and the IR activate several downstream signaling pathways including the MAPK and the PI3K/Akt pathways (20, 21). Activation of the MAPK and PI3K/Akt pathways by IGF1R/IR stimulation is associated with cell proliferation, survival, metabolism, protein synthesis, and cell growth in many cell types (22). Disruption of IGF/insulin signaling has been established as a cause of placental disruption that leads to FGR (23, 24). For example, *Igf1r* knockout mice exhibit a 55% reduction in birth weight when compared with WT mice (25). However, the regulation of IGF/insulin signaling components in the placenta requires further elucidation.

Although MAP3K4 has been shown to control early placental development during implantation, MAP3K4 regulation of growth-promoting pathways in the placenta and FGR is unknown. Herein, we report that inactivation of MAP3K4 kinase activity dysregulates the IGF1R/IR and Akt signaling axis, negatively affecting embryonic and placental growth, resulting in FGR. Surviving *Map3k4*^*KI/KI*^ adult mice were smaller in size and weight when compared with *Map3k4*^*WT/WT*^ littermates. *Map3k4*^*KI/KI*^ embryos and placentas displayed reduced size when compared with WT *Map3k4*^*WT/WT*^ embryos and placentas. Importantly, TS^KI^ cells, mature trophoblasts derived from the *in vitro* differentiation of TS^KI^ cells, and the *in vivo* tissues from *Map3k4*^*KI/KI*^ placentas exhibited disrupted IGF1R, IR, and Akt activity. Reduction in this pathway was primarily because of decreased expression of the IGF1R with MAP3K4 inactivation. We identified a previously unknown role for MAP3K4, histone acetyltransferase CREB-binding protein (CBP), and histone deacetylase 6 (HDAC6) in control of *Igf1r* transcript levels. In summary, we demonstrate that MAP3K4 regulates growth of the developing embryo and placenta by promoting the IGF1R/IR and Akt pathway in TS cells and placentas. Our data suggest that loss of MAP3K4 activity results in FGR, lethality prior to weaning, and reduced postnatal growth in surviving adults. These findings may lead to the development of clinically relevant therapeutic strategies to combat FGR in humans.

## Results

### Poor survival and reduced size of Map3k4^KI/KI^ mice when compared with Map3k4^*WT/WT*^ mice

MAP3K4 has previously been shown to be necessary for neurulation, skeletal patterning, and sex determination in mice (16, 18, 26). However, MAP3K4 kinase activity and its effect on embryonic and postnatal growth remains poorly understood. Previous studies by Shendy *et al.* (18) in a mixed 129/SvEv/C57BL/6N background that was backcrossed 1 time with C57BL/6N showed that *Map3k4*^*KI/KI*^ mice display male to female phenotypic sex reversal. Sex-reversed XY phenotypic female *Map3k4*^*KI/KI*^ mice had reduced weight at 6 months when compared with genotypic and phenotypic female *Map3K4*^*WT/WT*^ mice (18). These studies of adult *Map3K4*^*KI/KI*^ mice were limited to examination of XY phenotypic female *Map3k4*^*KI/KI*^ mice because of male gonadal sex reversal of the *Map3k4*^*KI/KI*^ males and embryonic lethality in the *Map3k4*^*KI/KI*^ females (18). To circumvent this issue, *Map3k4*^*KI/KI*^ mice were backcrossed to a pure 129/SvEv background and maintained in *Map3k4*^*WT/KI*^ heterozygote crosses. However, we observed only modest increases in survival rates. *Map3k4*^*KI/KI*^ mice in the 129/SvEv background still displayed a non-Mendelian ratio accounting for only 6% of the population compared with 4.5% in the mixed 129/SvEv/C57BL/6N background or 3.2% in the pure C57BL/6N background (Tables 1 and S1) (18). Sex-biased ratios were not observed in *Map3k4*^*KI/KI*^ mice on the pure 129/SvEv background as compared with a 4:1 female-biased sex ratio in the mixed background or the absence of *Map3k4*^*KI/KI*^ phenotypic males on the pure C57BL/6N background (Tables 1 and S1) (18). Furthermore, 100% of phenotypic female *Map3k4*^*KI/KI*^ mice examined in the 129/SvEv background (n = 6) presented with XX genotypes, suggesting that MAP3K4-dependent sex reversal was not occurring in the pure 129/SvEv background (Table 1).

**Table 1:** Reduced numbers of mice with homozygous deficiency in MAP3K4 kinase activity in the 129/SvEv background at weaning

*Map3k4* genotype	Male, n (%)	Female, n (%)	Total, n (%)
*WT/WT*	24 (14.37)	20 (11.98)	44 (26.35)
*WT/KI*	52 (31.13)	61 (36.53)	113 (67.66)
*KI/KI*	4 (2.40)	6 (3.59)^a^	10 (5.99)
Total	80 (47.9)	87 (52.1)	167 (100)

Data show mice sired over a 2-year period.

a Of the 6 phenotypic female *Map3k4*^*KI/KI*^ mice, all 6 were XX.

**Figure 1:**
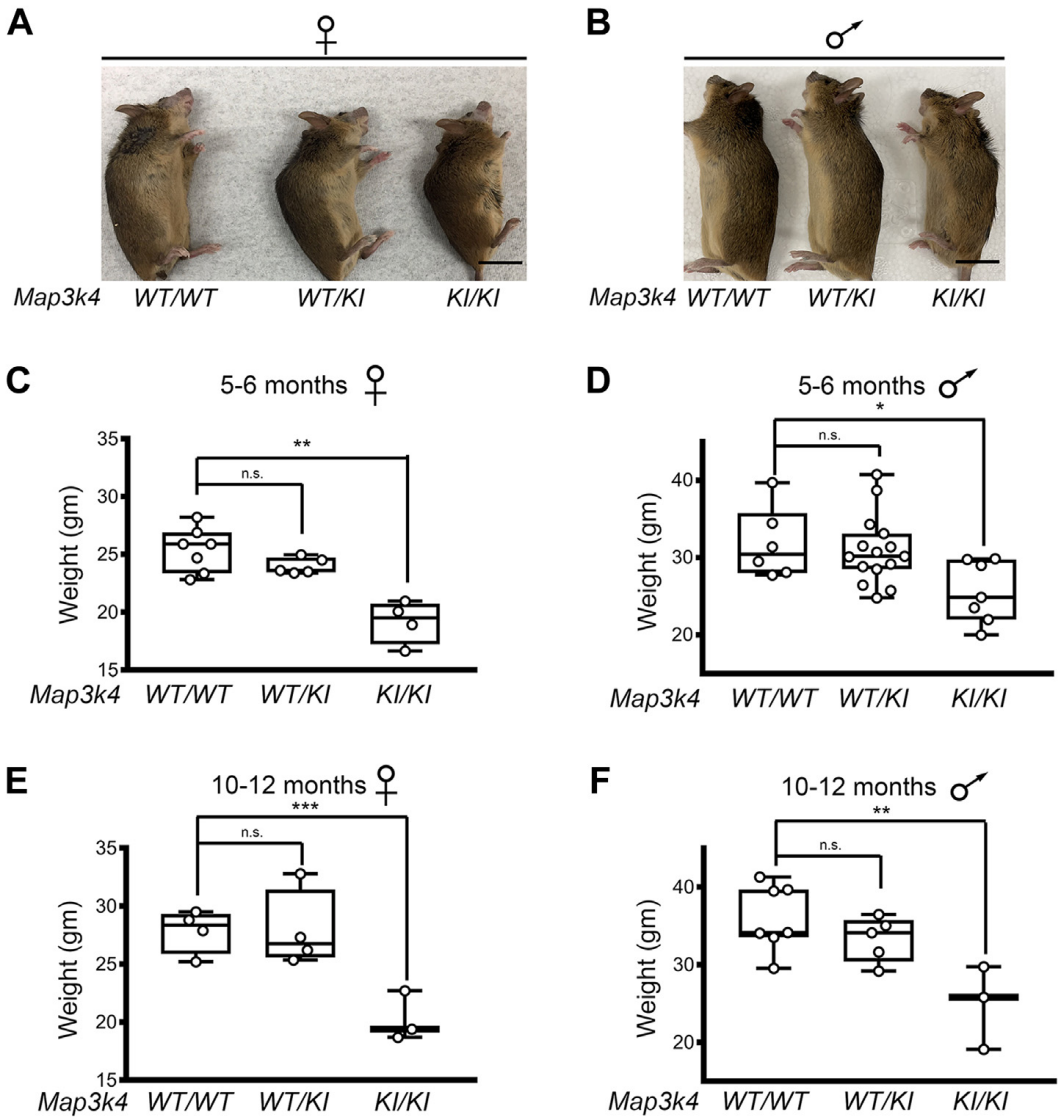
**Reduced bodyweight and size of *Map3k4***^***KI/KI***^**female and male 129/SvEv mice.***A* and *B*, body size of individual representative *Map3k4*^*WT/WT*^, *Map3k4*^*WT/KI*^, and *Map3k4*^*KI/KI*^ 129/SvEv mice aged 11 months in (*A*) females and (*B*) males. *Black* scale bar represents 2 cm. *C* and *D*, reduced average bodyweight of *Map3k4*^*KI/KI*^ mice compared with *Map3k4*^*WT/WT*^ mice aged 5 to 6 months in (*C*) females and (*D*) males. *E* and *F*, reduced average bodyweight of *Map3k4*^*KI/KI*^ mice compared with *Map3k4*^*WT/WT*^ mice aged 10 to 12 months in (*E*) females and (*F*) males. Data are displayed as *box plots*; each *dot* represents one individual. ∗*p* < 0.05; ∗∗*p* < 0.01; ∗∗∗*p* < 0.001; Student’s *t* test. KI, kinase inactive; *Map3k4*, mitogen-activated protein kinase kinase kinase 4; ns, not significant.

To further investigate the impact of MAP3K4 KI on long-term growth, we examined surviving adults in the pure 129/SvEv background. Our findings showed an overall reduced body size in both female (Fig. 1*A*) and male (Fig. 1*B*) *Map3k4*^*KI/KI*^ mice when compared with *Map3k4*^*WT/WT*^ mice. Next, mice were weighed at 5 to 6 months of age to determine if MAP3K4 KI resulted in reduced weight. Female *Map3k4*^*KI/KI*^ mice had a 26% reduction in weight, whereas male *Map3k4*^*KI/KI*^ mice had a 20% reduction in weight when compared with *Map3k4*^*WT/WT*^ mice (Fig. 1, *C* and *D*, Table S2). Importantly, this reduced weight was also observed at 10 to 12 months of age in both *Map3k4*^*KI/KI*^ females (27%) and males (31%) when compared with *Map3k4*^*WT/WT*^ mice (Fig. 1, *E* and *F*, Table S2). Taken together, these data show that KI of MAP3K4 results in reduced size and weight, suggesting MAP3K4 plays an important role in growth.

### Reduced embryonic and placental size of Map3k4^*KI/KI*^ conceptuses

We hypothesized that the reduced size observed in *Map3k4*^*KI/KI*^ adult mice was due in part to reduced growth during gestation. Careful examination of E13.5 embryos in the 129/SvEv/C57BL/6N mixed background revealed reduced crown-rump length of *Map3k4*^*KI/KI*^ embryos relative to littermate *Map3k4*^*WT/WT*^ embryos (Fig. S1, *A* and *B*). In addition, 71.4% of 129/SvEv/C57BL/6N *Map3k4*^*KI/KI*^ embryos displayed neural tube defects such as exencephaly (18). The liver is the first organ to be affected by FGR, and reduced liver size is commonly observed in fetuses and neonates displaying FGR (27, 28). Livers isolated at E13.5 from 129/SvEv/C57BL/6N *Map3k4*^*KI/KI*^ embryos exhibited reduced size when compared with *Map3k4*^*WT/WT*^ embryos (Fig. S1, *C* and *D*). Furthermore, we were unable to identify livers in 2 *Map3k4*^*KI/KI*^ embryos. Finally, because placental development is critical for embryonic maturation and growth, we investigated if placentas from *Map3k4*^*KI/KI*^ embryos were altered in size. Placental area was significantly reduced in the 129/SvEv/C57BL/6N *Map3k4*^*KI/KI*^ embryos, suggesting that MAP3K4 is involved in placental and fetal growth (Fig. S1, *E* and *F*).

**Figure 2:**
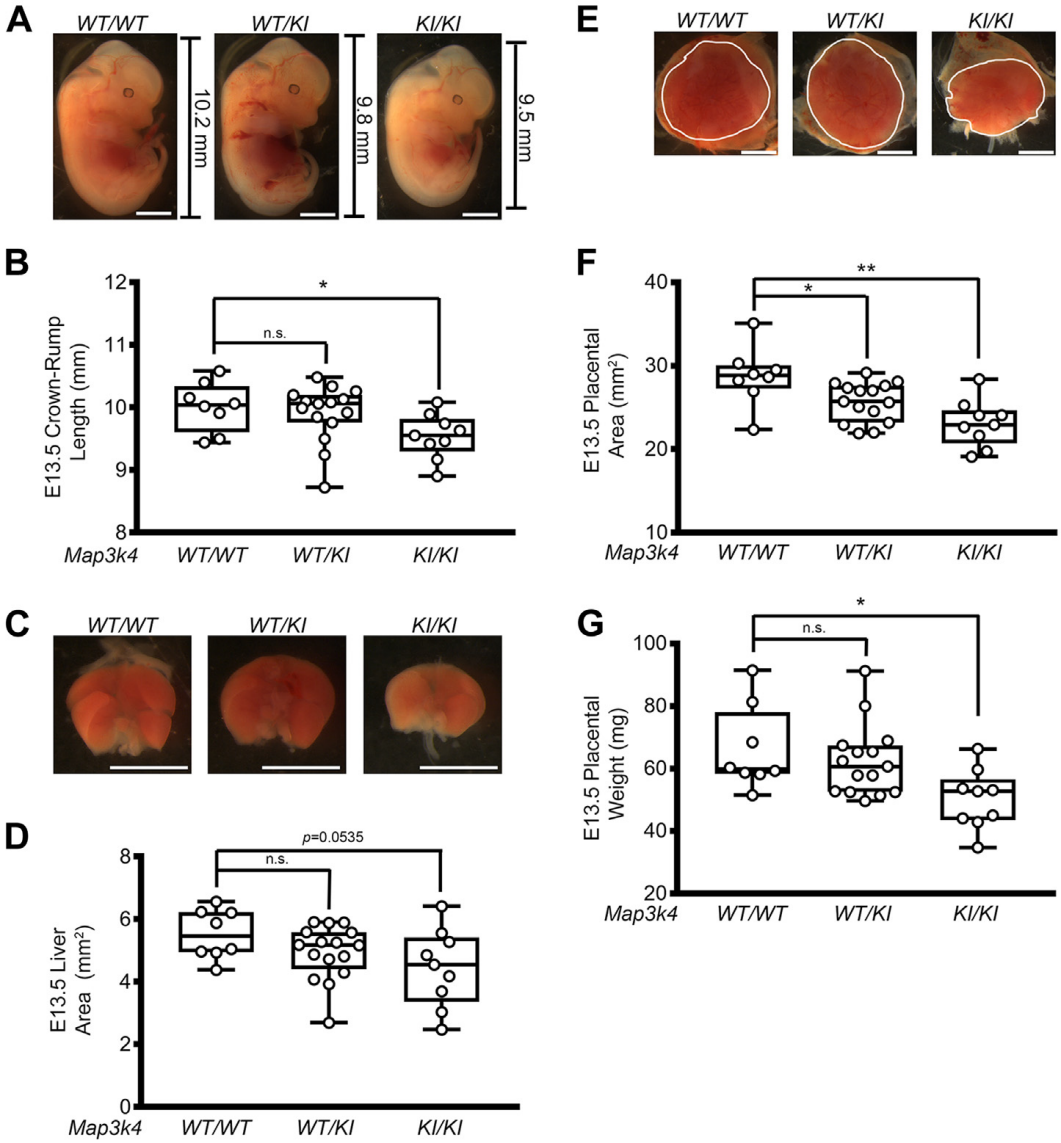
**MAP3K4 inactivation results in fetal growth restriction and decreased placental size in the 129/SvEv background.***A*, representative images of E13.5 *Map3k4*^*WT/WT*^, *Map3k4*^*WT/KI*^, and *Map3k4*^*KI/KI*^ embryos. *Black* scale bars and numbers indicate crown-rump length. *White* scale bar represents 2 mm. *B*, reduced crown-rump length of *Map3k4*^*KI/KI*^ E13.5 embryos compared with *Map3k4*^*WT/WT*^ E13.5 embryos. *C*, representative images of E13.5 *Map3k4*^*WT/WT*^, *Map3k4*^*WT/KI*^, and *Map3k4*^*KI/KI*^ livers. *White* scale bar represents 2 mm. *D*, liver area of *Map3k4*^*KI/KI*^ E13.5 embryos compared with E13.5 *Map3k4*^*WT/WT*^ embryos. *E*, representative images of E13.5 *Map3k4*^*WT/WT*^, *Map3k4*^*WT/KI*^, and *Map3k4*^*KI/KI*^ placentas outlined in *white*. *White* scale bar represents 2 mm. *F*, reduced placental area and (*G*) reduced placental weight of *Map3k4*^*KI/KI*^ E13.5 placentas compared with *Map3k4*^*WT/WT*^ E13.5 placentas. *B*, *D*, *F*, and *G*, data are displayed as *box plots*; each *dot* represents one individual. ∗*p* < 0.05; ∗∗*p* < 0.01; Student’s *t* test. KI, kinase inactive; MAP3K4, mitogen-activated protein kinase kinase kinase 4; ns, not significant.

Based on these findings in a mixed genetic background, we examined *Map3k4*^*KI/KI*^ embryos at E13.5 in the pure 129/SvEv background. In contrast to the mixed background where 50% of *Map3k4*^*KI/KI*^ embryos were lost by E13.5 (18), *Map3k4*^*KI/KI*^ embryos in the pure 129/SvEv background were present in Mendelian numbers with 22.22% being homozygous for the MAP3K4 KI mutation (Table 2). These data suggest that the loss of *Map3k4*^*KI/KI*^ individuals occurs later in the pure 129/SvEv background. Similar to the mixed background, the crown-rump length of E13.5 *Map3k4*^*KI/KI*^ embryos in the 129/SvEv background was significantly reduced when compared with *Map3k4*^*WT/WT*^ embryos (Fig. 2, *A* and *B*, Table S3). *Map3k4*^*KI/KI*^ livers isolated at E13.5 were also reduced in size when compared with *Map3k4*^*WT/WT*^ livers, but these differences did not reach statistical significance (*p* = 0.0535) (Fig. 2, *C* and *D*; Table S3). Finally, *Map3k4*^*KI/KI*^ placentas showed a 20% reduction in total area and a 24% reduction in weight when compared with *Map3k4*^*WT/WT*^ placentas (Fig. 2, *E*–*G*, Table S3). Taken together, these data show that MAP3K4 KI results in embryonic growth restriction, and this reduction in size correlates with reduced placental size. These data suggest that MAP3K4 controls embryonic and placental growth, and the loss of MAP3K4 activity results in FGR.

**Table 2:** Mendelian numbers of *Map3k4*^*KI/KI*^ KI 129/SvEv embryos at E13.5

*Map3k4* genotype	Percent of total embryos	Percent with neural tube defects	n of embryos
*WT/WT*	20.37	0	11
*WT/KI*	57.41	0	31
*KI/KI*	22.22	25	12
Total	100	NA	54

NA, not applicable.

Six resorptions were detected.

### Altered activation of receptor tyrosine kinases in MAP3K4 KI TS cells

**Figure 3:**
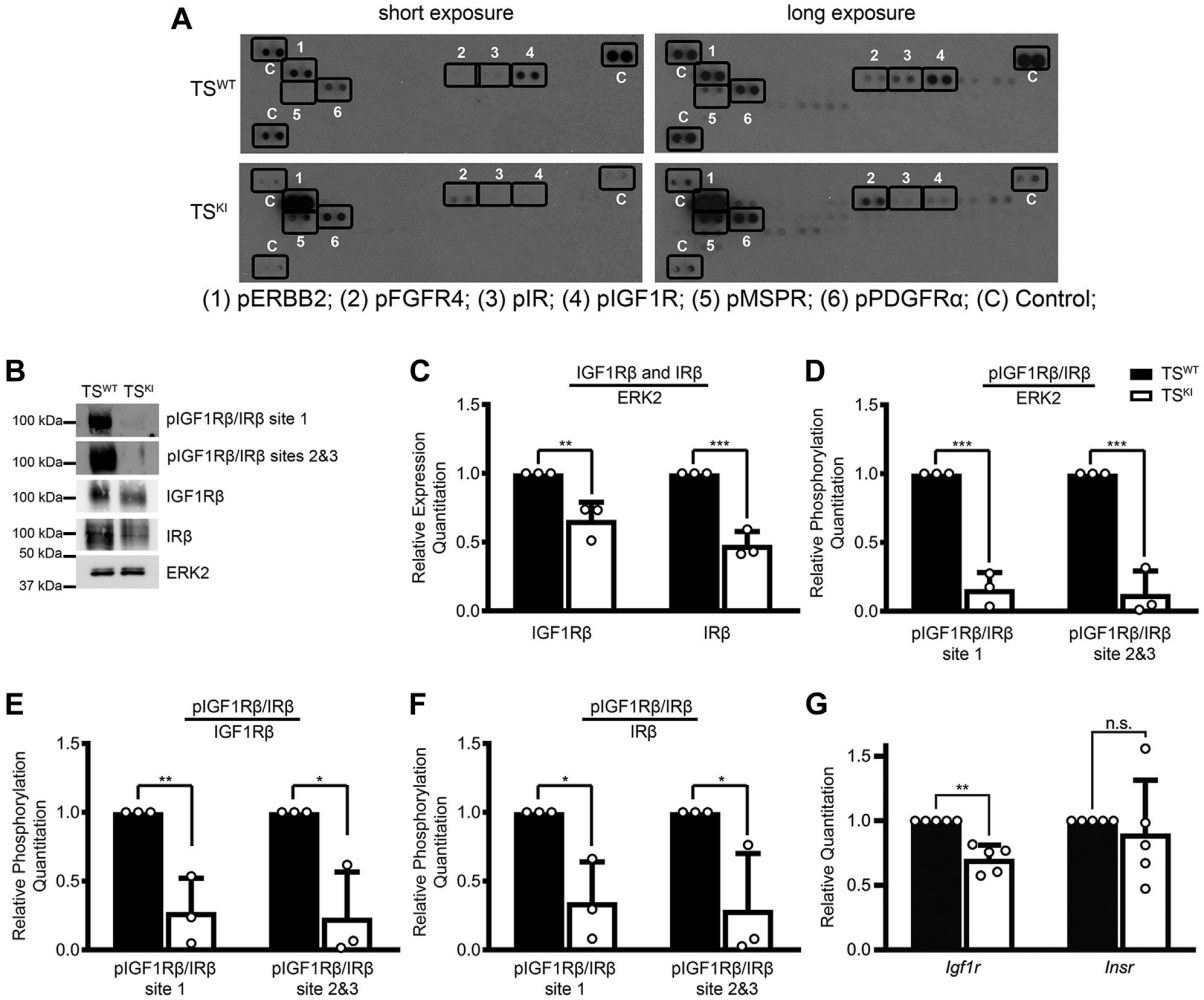
**MAP3K4 kinase inactivation results in reduced expression and basal phosphorylation of the IGF1R and IR.***A*, phospho-RTK array of TS^WT^ and TS^KI^ cells showing tyrosine phosphorylation of 39 different RTKs spotted in duplicate shown at either short or long exposure times. Each blot has 3 pairs of internal controls (*C*). *B*, kinase inactivation of MAP3K4 in TS cells results in reduced total and phosphorylated IGF1Rβ and IRβ. Site 1 represents pIGF1Rβ Tyr1131 and pIRβ Tyr1146, site 2 represents IGF1Rβ Tyr1135 and pIRβ Tyr1150, and site 3 represents pIGF1Rβ Tyr1136 and pIRβ Tyr1151. *C*–*F*, densitometry was used to quantify 3 biologically independent experiments. Expression was normalized to ERK2 (*C*), and phosphorylation was normalized to ERK2 (*D*), total IGF1Rβ (*E*), or total IRβ (*F*). Data show the mean ± SD of 3 biologically independent experiments. *G*, *Igf1r* transcripts are reduced in TS^KI^ cells relative to TS^WT^ cells. qPCR data normalized to *Rps11* are expressed as a fold change relative to TS^WT^ cells and are the mean ± SD of five biologically independent experiments. *A* and *B*, images are representative of 3 biologically independent experiments. ∗*p* < 0.05; ∗∗*p* < 0.01; ∗∗∗*p* < 0.001; Student’s *t* test. ERK2, extracellular-regulated kinase 2; IGF1R, insulin-like growth factor 1 receptor; IR, insulin receptor; KI, kinase inactive; MAP3K4, mitogen-activated protein kinase kinase kinase 4; ns, not significant; qPCR, quantitative PCR; RTK, receptor tyrosine kinase; TS, trophoblast stem.

TS cells are isolated from the TE of the developing blastocyst and give rise to all trophoblast cell types of the placenta (14). Activation of receptor tyrosine kinases (RTKs) in the placenta is necessary to promote embryonic and placental growth and survival (29, 30). Because of the reduced size of *Map3k4*^*KI/KI*^ embryos and placentas, and the postweaning growth deficiency in adult mice, we examined RTK activation in TS cells isolated from *Map3K4*^*KI/KI*^ embryos (TS^KI^). Using RTK arrays to examine tyrosine phosphorylation of 39 different RTKs, we found that the phosphorylation of 2 members of the IR family, IR and IGF1R, was reduced in TS^KI^ cells when compared with TS^WT^ cells (Fig. 3*A*, position #3, long exposure, and position #4, long and short exposures). In contrast, phosphorylation of the proto-oncogene and growth promoting Erb-B2 receptor tyrosine kinase 2 (ERBB2) and the fibroblast growth factor receptor 4 (FGFR4) were increased in TS^KI^ cells when compared with TS^WT^ cells (Fig. 3*A*, positions #1 and #2). The IGF and insulin signaling pathways have been previously shown to regulate fetal growth and placental development, but the involvement of MAP3K4 in regulating growth or the IGF1R and IR was unknown (31, 32). IGF1R and IR homodimer or heterodimer activation occurs upon the binding of ligand, either insulin, IGF1, or IGF2. Ligand binding induces a conformational change promoting autophosphorylation of 3 tyrosine residues in the kinase activation loop located on the transmembrane β subunit (33, 34). To examine IGF1R and IR phosphorylation, we utilized a commercially available phospho-specific antibody, which detects pIGF1Rβ Tyr1131 and pIRβ Tyr1146 (site 1). A separate phospho-specific antibody was used to detect both pIGF1Rβ Tyr1135 and pIRβ Tyr1150 (site 2) and pIGF1Rβ Tyr1136 and pIRβ Tyr1151 (site 3). Western blotting with these antibodies showed that IGF1Rβ and IRβ phosphorylation was greatly reduced in TS^KI^ cells when compared with TS^WT^ cells, confirming the RTK array results (Fig. 3*B*). Western blotting with antibodies for total IGF1Rβ and IRβ showed decreased expression of both these proteins (Fig. 3*B*). Densitometry analyses with normalization to extracellular-regulated kinase 2 (ERK2) showed statistically significant decreases in both total IGF1Rβ and IRβ expression (Fig. 3*C*). Because the phospho-specific antibodies simultaneously detect both IGF1Rβ and IRβ, we performed densitometry analyses of receptor phosphorylation compared with ERK2, IGF1Rβ, IRβ, or IGF1Rβ and IRβ. Phosphorylation of the IGF1Rβ and IRβ was reduced in TS^KI^ cells relative to TS^WT^ cells in all comparisons (Fig. 3, *D*–*F* and Fig. S2). However, reductions in receptor phosphorylation were more significant when normalized to ERK2 *versus* the IGF1Rβ and IRβ, suggesting that reduced IGF1Rβ and IRβ phosphorylation in TS^KI^ cells was due in part to reductions in total IGF1Rβ and IRβ protein (c.f., Fig. 3, *D–E* and *F*). Because of the reduced IGF1Rβ and IRβ protein levels, we measured *Igf1r* and *Insr* transcript levels, finding that only *Igf1r* transcript levels were decreased in TS^KI^ cells when compared with TS^WT^ cells, as measured by quantitative PCR (qPCR; Fig. 3*G*). The ratio of decreased *Igf1r* transcript to decreased protein was 1.06, suggesting that reduced IGF1R protein was due to decreased transcript expression. Together, these data suggest that total expression levels and thus basal phosphorylation of the IGF1R and IR are dependent on MAP3K4 kinase activity.

### Reduced activation of signaling pathways controlled by IGF1R and IR activity

**Figure 4:**
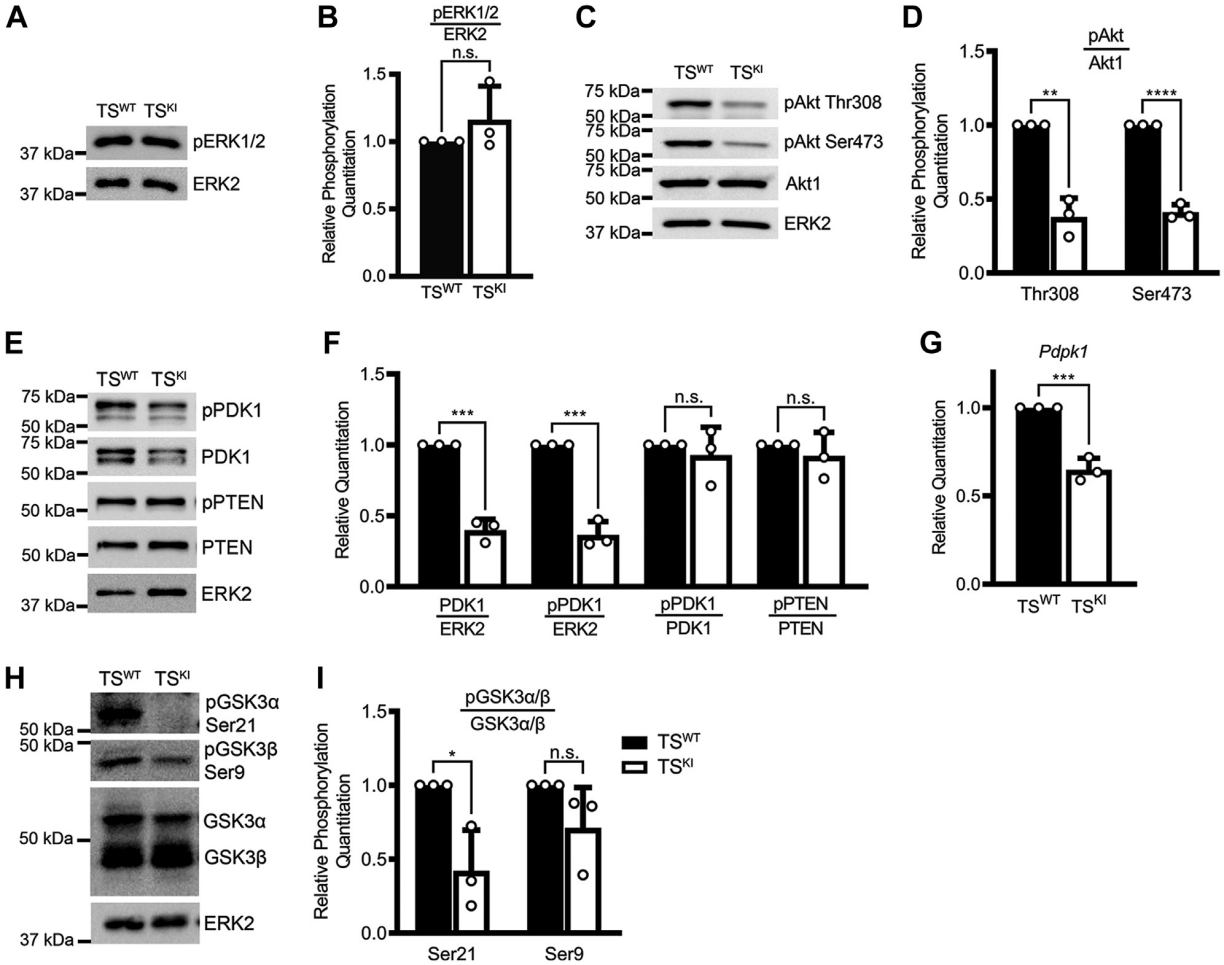
**The Akt signaling pathway is dependent on MAP3K4 kinase activity.***A*, ERK1/2 phosphorylation is unchanged with MAP3K4 kinase inactivation. *B*, densitometry analyses are shown with phosphorylation normalized to ERK2. *C*, MAP3K4 kinase inactivation in TS cells results in reduced Akt phosphorylation. *D*, densitometry analyses are shown with phosphorylation normalized to Akt1. *E*, PDK1 expression and phosphorylation at Ser241 are decreased with MAP3K4 inactivation. *F*, densitometry analyses are shown. PDK1 expression was normalized to ERK2. PDK1 phosphorylation was normalized to ERK2 or PDK1. PTEN inhibitory phosphorylation at Ser380/Thr382/Thr383 was normalized to PTEN protein levels. *G*, *Pdpk1* transcripts are reduced in TS^KI^ cells relative to TS^WT^ cells. qPCR data normalized to *Rps11* are expressed as a fold change relative to TS^WT^ cells and are the mean ± SD of 3 biologically independent experiments. *H*, GSK3α phosphorylation is reduced with MAP3K4 inactivation. *I*, densitometry analyses are shown. Phosphorylated GSK3α was normalized to total GSK3α protein, and phosphorylated GSK3β was normalized to total GSK3β protein. *A*, *C*, *E*, and *H*, images are representative of 3 biologically independent experiments. *B*, *D*, *F*, and *I*, densitometry was used to quantify 3 biologically independent experiments. Data shown are the mean ± SD. ∗*p* < 0.05; ∗∗*p* < 0.01; ∗∗∗*p* < 0.001; ∗∗∗∗*p* < 0.0001; Student’s *t* test. Akt, protein kinase B; ERK1/2, extracellular-regulated kinase 1/2; GSK3α, glycogen synthase kinase 3 alpha; KI, kinase inactive; MAP3K4, mitogen-activated protein kinase kinase kinase 4; ns, not significant; PDK1, 3-phosphoinositide-dependent protein kinase 1; PTEN, phosphatase and tensin homolog; qPCR, quantitative PCR; TS, trophoblast stem.

IGF1R/IR signaling activates several pathways including MAPK and Akt pathways (21). Based on our data showing TS^KI^ cells had reduced IGF1R and IR protein expression and phosphorylation, we predicted that phosphorylation of IGF1R/IR downstream effectors might also be decreased. To test this hypothesis, we performed Western blot analyses using a phospho-specific antibody for the MAPK ERK1 and ERK2 in TS^WT^ and TS^KI^ cells. Basal phosphorylation of ERK1/2 was similar in the TS^KI^ and TS^WT^ cells (Fig. 4, *A* and *B*). These data show that MAP3K4 KI does not affect ERK phosphorylation in TS cells.

The Akt signaling network is activated by IGF1R/IR signaling and promotes cell growth and survival (21, 22). Akt activity is induced by phosphorylation at 2 sites, threonine 308 (Thr308) and serine 473 (Ser473). Although partial Akt activation occurs with single Thr308 phosphorylation, full activity requires dual Thr308 and Ser473 phosphorylation (22). Akt phosphorylation at both Thr308 and Ser473 was greatly diminished in TS^KI^ cells when compared with TS^WT^ cells (Fig. 4, *C* and *D*). The IGF1R/IR pathway activates 3-phosphoinositide-dependent protein kinase 1 (PDK1), which directly phosphorylates and activates Akt at Thr308 (35). PDK1 activation requires the phosphorylation of Ser241 in the PDK1 active site; however, IGF signaling does not induce phosphorylation of this site (36). PDK1 Ser241 phosphorylation and total PDK1 expression normalized to total ERK2 protein were reduced in TS^KI^ cells when compared with TS^WT^ cells, consistent with reduced phosphorylation of Akt at Thr308 (Fig. 4, *E* and *F*). However, phospho-PDK1 normalized to total PDK1 levels were not different between TS^WT^ and TS^KI^ cells, indicating that the reduction of phosphorylated PDK1 was due to lower protein expression of PDK1, similar to the IGF1R and IR (Fig. 4, *E* and *F*). Based on the reduced protein expression of PDK1, we measured transcript levels, finding reduced *Pdpk1* transcript in TS^KI^ cells when compared with TS^WT^ cells (Fig. 4*G*). The ratio of reduced *Pdpk1* transcript to protein was 1.63, suggesting that reduced PDK1 protein was due to both the loss of *Pdpk1* transcript and disruption of protein. Phosphatase and tensin homolog (PTEN) acts as a negative regulator of the Akt pathway by dephosphorylating phosphatidylinositol (3,4,5)-trisphosphate and disrupting Akt localization to the plasma membrane, preventing Akt phosphorylation and activation by PDK1 (37). To determine if the reduction of Akt phosphorylation was PTEN dependent, we probed for total and phospho-PTEN levels. The phospho-specific antibody detects the phosphorylation of PTEN at Ser380/Thr382/Thr383; phosphorylation of these sites inactivates PTEN (38, 39). Total and phosphorylated PTEN levels were similar between TS^WT^ and TS^KI^ cells, suggesting that the reduction of phosphorylated Akt was independent of changes in PTEN expression and activity (Fig. 4, *E* and *F*). In addition to changes in upstream regulation of Akt by PDK1, TS^KI^ cells had reduced phosphorylation of the Akt substrate, glycogen synthase kinase 3 alpha (GSK3α) at serine 21 relative to TS^WT^ cells, suggesting Akt kinase activity is reduced in TS^KI^ cells (Fig. 4, *H* and *I*). Furthermore, TS^KI^ cells displayed partial reduction of glycogen synthase kinase 3 beta (GSK3β) phosphorylation when compared with TS^WT^ cells; however, these changes were not statistically significant (Fig. 4, *H* and *I*). Taken together, these data demonstrate that the Akt pathway is inhibited in TS cells lacking MAP3K4 kinase activity.

### Examination of potential compensation for loss of IGF1R/IR activity by ERBB2 and FGFR4

**Figure 5:**
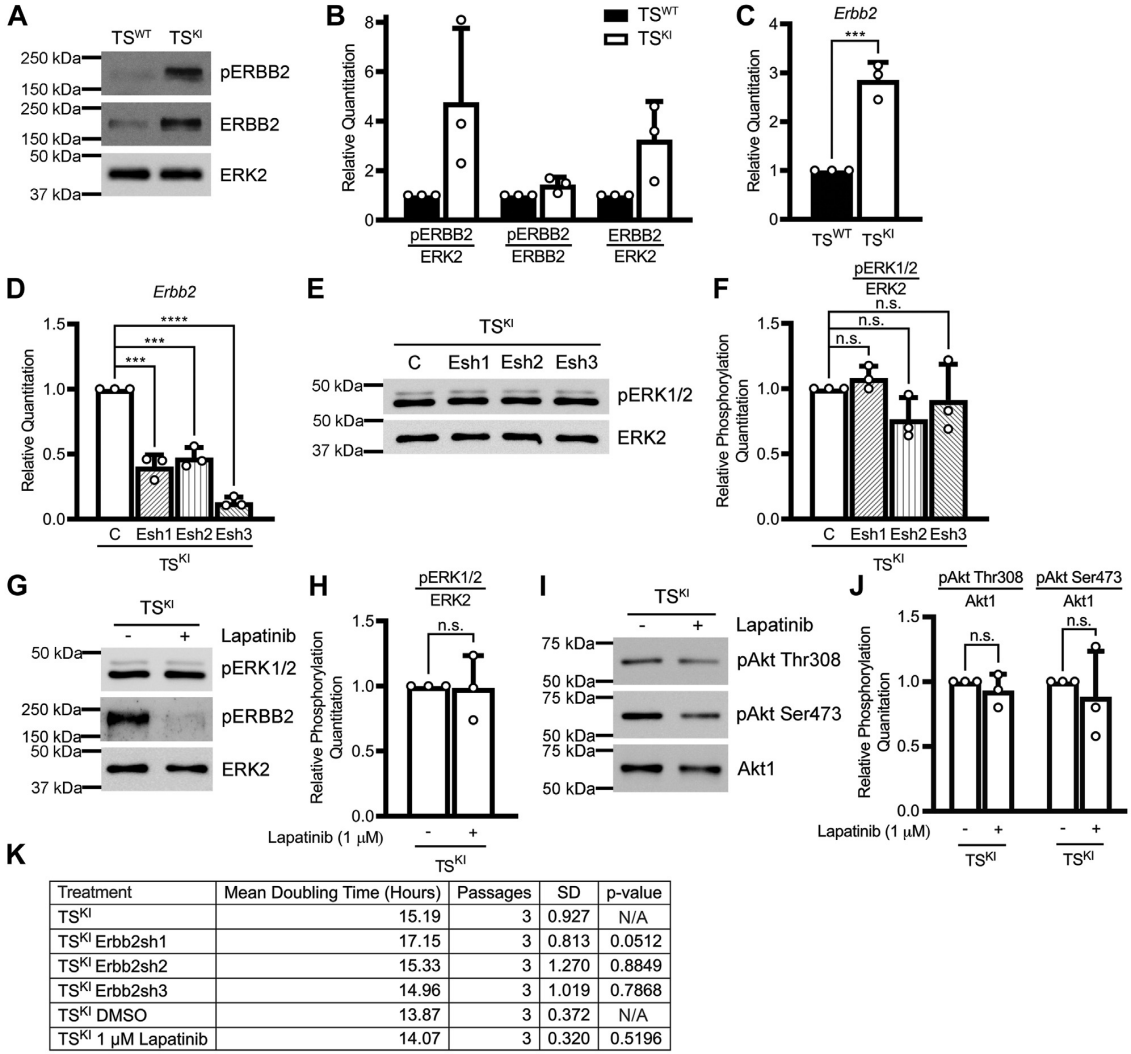
**ERBB2 does not compensate for effects of reduced IGF1R/IR signaling on Akt or ERK phosphorylation in TS**^**KI**^**cells.***A*, increased phosphorylation and expression of ERRB2 in TS cells lacking MAP3K4 kinase activity. *B*, densitometry analyses are shown. *C*, increased *Erbb2* transcript in TS^KI^ cells relative to TS^WT^ cells. Quantitative PCR (qPCR) data normalized to *Rps11* are expressed as a fold change relative to TS^WT^ cells and are the mean ± SD of 3 biologically independent experiments. *D*, reduced *Erbb2* expression through *Erbb2* shRNA knockdown in TS^KI^ cells with 3 independent shRNAs, Esh1, Esh2, and Esh3. qPCR data normalized to *Rps11* are expressed as a fold change relative to TS^KI^ cells and are the mean ± SD of 3 biologically independent experiments. *E*, *Erbb2* shRNA knockdown in TS^KI^ cells does not affect ERK1/2 phosphorylation. *F*, densitometry analyses normalizing phosphorylated ERK1/2 to total ERK2 protein. *G*–*J*, cells were treated with either vehicle control (DMSO) or 1 μM lapatinib for 48 h. Inhibition of ERBB2 with lapatinib does not affect ERK1/2 phosphorylation or Akt phosphorylation. *H*, densitometry analyses normalized phosphorylated ERK1/2 to total ERK2 protein. *J*, densitometry analyses normalized phosphorylated Akt to Akt1. *K*, *Erbb2* shRNA knockdown or ERBB2 inhibition with lapatinib does not significantly affect cellular doubling time. *A*, *E*, *G*, and *I*, images are representative of 3 biologically independent experiments. *B*, *F*, *H*, and *J*, data shown are the mean ± SD of densitometry analyses of 3 biologically independent experiments. ∗∗∗*p* < 0.001; ∗∗∗∗*p* < 0.0001; Student’s *t* test. ns, not significant. Akt, protein kinase B; DMSO, dimethyl sulfoxide; ERBB2, Erb-B2 receptor tyrosine kinase 2; ERK, extracellular-regulated kinase; IGF1R, insulin-like growth factor 1 receptor; IR, insulin receptor; MAP3K4, mitogen-activated protein kinase kinase kinase 4; TS, trophoblast stem.

Fig. 3*A* showed increased phosphorylation of growth-promoting RTKs, ERBB2, and FGFR4, in TS^KI^ cells relative to TS^WT^ cells. Both ERBB2 and FGFR4 have been shown to activate ERK and/or Akt in some contexts such as cancer (40, 41). Based on these previous studies, we hypothesized a possible role for these receptors in promoting the growth of TS^KI^ cells by activating ERK and/or by providing compensation for reduced activation of the Akt pathway. We confirmed increased ERBB2 phosphorylation in TS^KI^ cells using ERBB2 phospho-specific antibodies showing results similar to the RTK array (Fig. 5, *A* and *B*). Interestingly, total ERBB2 transcript and protein were also elevated in TS^KI^ cells when compared with TS^WT^ cells (Fig. 5, *A*–*C*). To determine a potential role for ERBB2 in TS^KI^ cells, we performed lentiviral shRNA knockdown of *Erbb2* in TS^KI^ cells using 3 independent shRNAs that reduced *Erbb2* transcript between 53 and 87% when compared with control-infected TS^KI^ cells (Fig. 5*D*). shRNA knockdown of *Erbb2* did not alter the phosphorylation of ERK, and AKT phosphorylation remained difficult to detect (Fig. 5, *E* and *F*, data not shown). In addition to altering *Erbb2* expression, we examined the impact of 48-h treatment with the EGF1R/ERBB2 inhibitor lapatinib on ERK and Akt phosphorylation. Lapatinib was sufficient to inhibit the phosphorylation of ERBB2 but had no significant effects on phosphorylation of ERK or Akt (Fig. 5, *G*–*J*). Furthermore, cellular doubling time was not significantly altered by either shRNA knockdown of *Erbb2* or inhibition with lapatinib (Fig. 5*K*). Together, these data do not support a role for ERBB2 in compensation for reduced Akt activity in TS^KI^ cells.

**Figure 6:**
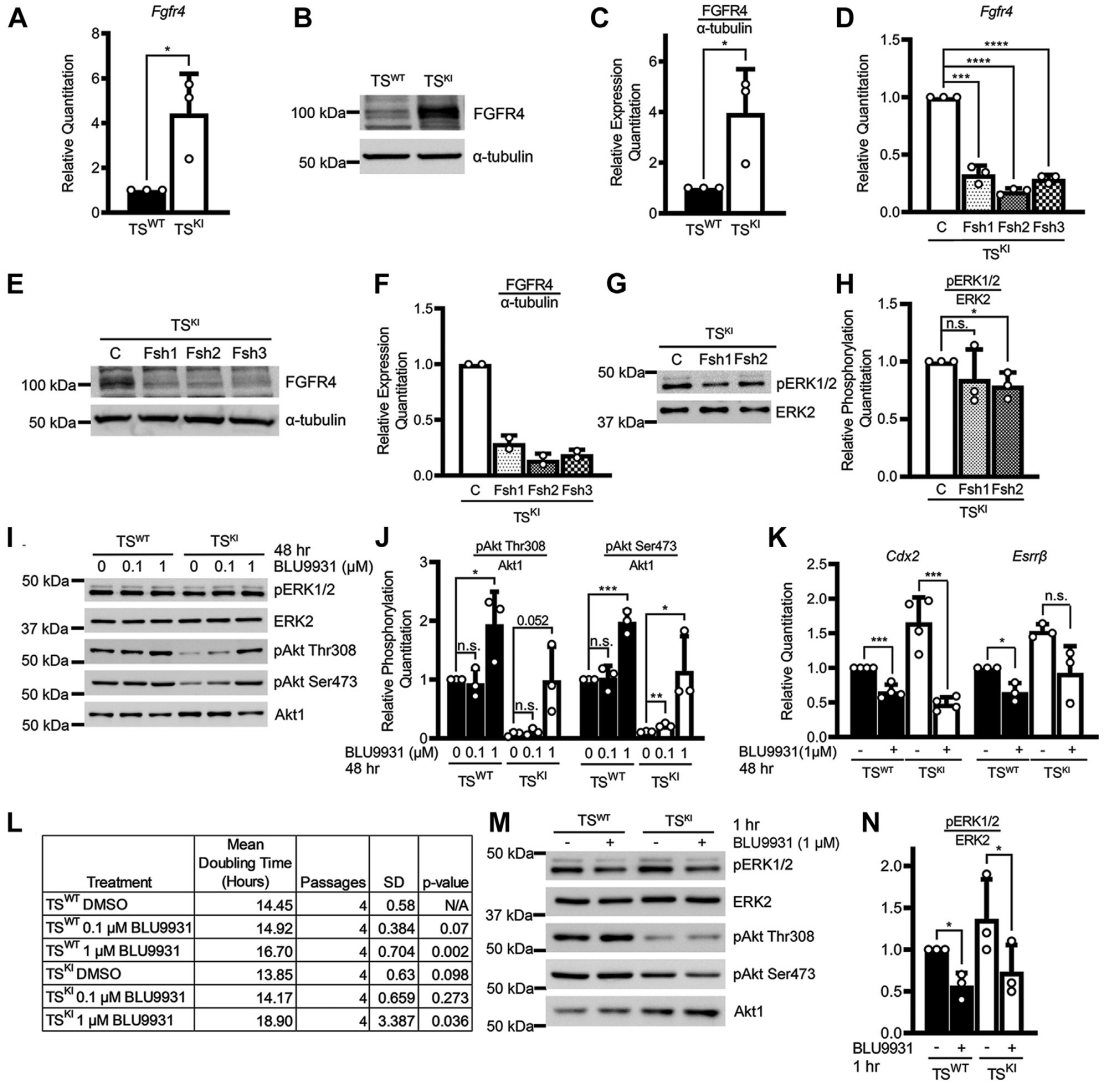
**FGFR4 promotes ERK1/2 phosphorylation and maintenance of stemness in trophoblast stem cells.***A*–*C*, *Fgfr4* transcript and protein are increased in TS^KI^ cells relative to TS^WT^ cells. *A*, qPCR data normalized to *Rps11* are expressed as a fold change relative to TS^WT^ cells and are the mean ± SD of 3 biologically independent experiments. *B*, Western blotting images and (*C*) densitometry analyses with normalization of FGFR4 to α-tubulin are shown. *D*, reduced *Fgfr4* expression through shRNA knockdown in TS^KI^ cells. qPCR data normalized to *Rps11* are expressed as a fold change relative to TS^KI^ cells and are the mean ± SD of 3 biologically independent experiments. *E* and *F*, reduced FGFR4 protein with shRNA knockdown of *Fgfr4*. Data show the mean ± range of 2 biologically independent experiments. *G* and *H*, ERK1/2 phosphorylation is not consistently affected by *Fgfr4* shRNA knockdown. *I*–*N*, cells were treated with either vehicle control (DMSO) or the indicated concentration of BLU9931. *I* and *J*, chronic inhibition of FGFR4 with BLU9931 for 48 h increases Akt phosphorylation. *K*, chronic BLU9931 treatment for 48 h inhibits the expression of markers of TS cell stemness. qPCR data normalized to *Rps11* are expressed as a fold change relative to TS^WT^ cells and are the mean ± SD of 4 (*Cdx2*) or 3 (*Esrrb*) biologically independent experiments. *L*, chronic BLU9931 treatment for 48 h reduced cellular doubling time. *M* and *N*, acute inhibition of FGFR4 by treatment with BLU9931 for 1 h inhibits ERK1/2 phosphorylation. *B*, *G*, *I*, and *M*, Western blotting images are representative of 3 biologically independent experiments. *C*, *H*, *J*, and *N*, densitometry analyses shown are the mean ± SD of 3 biologically independent experiments. ∗*p* < 0.05; ∗∗*p* < 0.01; ∗∗∗*p* < 0.001; ∗∗∗∗*p* < 0.0001; Student’s *t* test. Akt, protein kinase B; DMSO, dimethyl sulfoxide; ERK1/2, extracellular-regulated kinase 2; FGFR4, fibroblast growth factor receptor 4; KI, kinase inactive; ns, not significant; qPCR, quantitative PCR; TS, trophoblast stem.

We also examined a potential role for FGFR4 in compensation for loss of the IGF1R/IR. Because of the lack of commercially available phospho-specific antibodies that detect endogenous FGFR4, we were unable to confirm the increases in FGFR4 phosphorylation. However, *Fgfr4* transcript and protein expression were increased in TS^KI^ cells relative to TS^WT^ cells (Fig. 6, *A*–*C*). Lentiviral shRNA knockdown of *Fgfr4* using 3 independent shRNAs reduced FGFR4 expression (Fig. 6, *D–F*). Under these conditions, phosphorylation of ERK was only modestly changed and not consistent with the relative reduction in FGFR4 levels for each shRNA (Fig. 6, *G* and *H*). Similar to control-infected TS^KI^ cells, phosphorylation of Akt in TS^KI^ cells with *Fgfr4* knockdown remained nearly undetectable, and cellular doubling time was not consistently affected by *Fgfr4* shRNA knockdown in TS^KI^ cells (Fig. S3*A*, data not shown). Because the reduction of *Fgfr4* by shRNA knockdown was incomplete, we also examined the impact of treatment with the FGFR4 selective inhibitor BLU9931 (42). ERK phosphorylation was not altered by chronic treatment with BLU9931 for 48 h (Figs. 6*I* and S3*B*). In contrast, phosphorylation of Akt was strongly induced by FGFR4 inhibition in a dose-dependent manner (Fig. 6, *I* and *J*). These findings of increased Akt phosphorylation after chronic inhibition of FGFR4 do not support a role for FGFR4 in compensation for loss of Akt phosphorylation in TS^KI^ cells. Examination of TS cell markers revealed that FGFR4 inhibition resulted in statistically significant decreases in *Cdx2* expression (Fig. 6*K*). Consistent with reduced stemness marker expression, FGFR4 inhibition with BLU9931 lengthened the doubling time (Fig. 6*L*). However, these effects were only observed with high doses of BLU9931, suggesting that chronic drug treatment might be inducing alternative signaling pathways to promote survival (Fig. 6*L*). Based on these findings, we examined the impact of acute treatment with BLU9931 for 1 h on phosphorylation of ERK and Akt. Surprisingly, BLU9931 treatment for 1 h was sufficient to reduce phosphorylation of ERK but had no effect on Akt phosphorylation (Figs. 6, *M* and *N*, and S3*C*). Taken together, these data suggest that TS^KI^ cells have increased FGFR4 expression that may function to promote stemness in TS^KI^ cells. However, these data do not support a role for FGFR4 in compensation for reduced IGF1R and IR activation or the loss of Akt phosphorylation.

### Decreased responsiveness of stem cells lacking MAP3K4 kinase activity to insulin stimulation because of reduced expression of the IGF1R and IR

**Figure 7:**
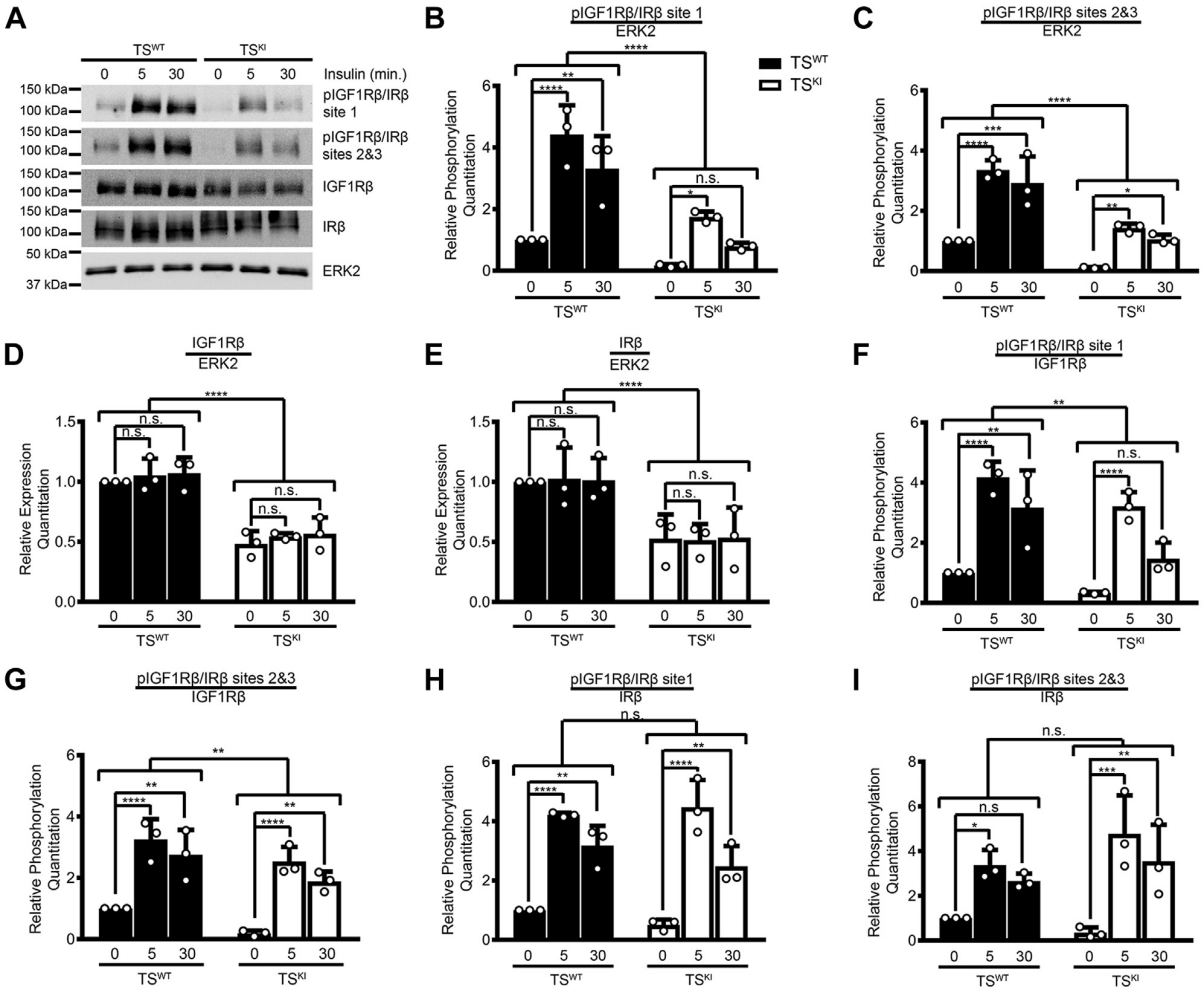
**MAP3K4 kinase inactivation results in reduced insulin-stimulated phosphorylation of the IGF1R and IR because of reduced IGF1R and IR expression.***A*, kinase inactivation of MAP3K4 in TS cells results in reduced IGF1Rβ and IRβ expression and phosphorylation. Site 1 represents pIGF1Rβ Tyr1131 and pIRβ Tyr1146, site 2 represents IGF1Rβ Tyr1135 and pIRβ Tyr1150, and site 3 represents pIGF1Rβ Tyr1136 and pIRβ Tyr1151. Western blot image is representative of 3 biologically independent experiments. *B*–*I*, densitometry was used to quantify 3 biologically independent experiments. Graphs show the mean ± SD. *B* and *C*, phosphorylation was normalized to ERK2. *D* and *E*, expression was normalized to ERK2. *F* and *G*, phosphorylation was normalized to IGF1Rβ. *H* and *I*, phosphorylation was normalized to IRβ. ∗*p* < 0.05; ∗∗*p* < 0.01; ∗∗∗*p* < 0.001; ∗∗∗∗*p* < 0.0001; Student’s *t* test; two-way ANOVA. ERK2, extracellular-regulated kinase 2; IGF1R, insulin-like growth factor 1 receptor; IR, insulin receptor; MAP3K4, mitogen-activated protein kinase kinase kinase 4; ns, not significant; TS, trophoblast stem; Insulin, 25 μg/ml.

Based on the lack of strong evidence for compensation by ERBB2 or FGFR4 on the Akt pathway in TS^KI^ cells, we focused on the IGF1R and IR. We examined ligand-stimulated phosphorylation of the IGF1R/IR. Because of the requirement of serum to maintain TS cell stemness, all experiments were performed on unstarved stem cells. Although insulin and IGF have higher affinities for the IR and IGF1R, respectively, insulin can activate both the IGF1R and IR (43). TS^WT^ and TS^KI^ cells were treated with a high dose of insulin (25 μg/ml) and probed for phosphorylated IGF1Rβ and IRβ (Fig. 7*A*). TS^WT^ cells had robust insulin-stimulated activation of IGF1Rβ/IRβ at both time points, showing an increase in all 3 autophosphorylation sites when compared with control-treated TS^WT^ cells (Fig. 7*A*). In contrast, the response of TS^KI^ cells was severely blunted. When normalized to total ERK2 levels, TS^KI^ cells showed a significant reduction in response to insulin treatment (Fig. 7, *B* and *C*). However, similar to measurements performed under basal conditions (Fig. 3), total IGF1Rβ and IRβ protein expression were also reduced in insulin-stimulated TS^KI^ cells relative to TS^WT^ cells (Fig. 7, *D* and *E*). Although a two-way ANOVA showed statistically significant differences overall between TS^WT^ and TS^KI^ cell sample groups, normalization of phosphorylation levels to either total IGF1Rβ, IRβ, or combined IGF1Rβ and IRβ protein showed that insulin-stimulated phosphorylation of IGF1Rβ/IRβ was similar between TS^WT^ and TS^KI^ cells (Figs. 7, *F*–*I* and S4, *A* and *B*). Taken together, these data indicate that biologically relevant mechanisms such as extracellular ligand stimulation of the IGF1R and IR were impaired in TS cells with MAP3K4 KI, as even responses to high doses of insulin were diminished. However, this reduced responsiveness to insulin was due to MAP3K4 control of IGF1R and IR expression.

**Figure 8:**
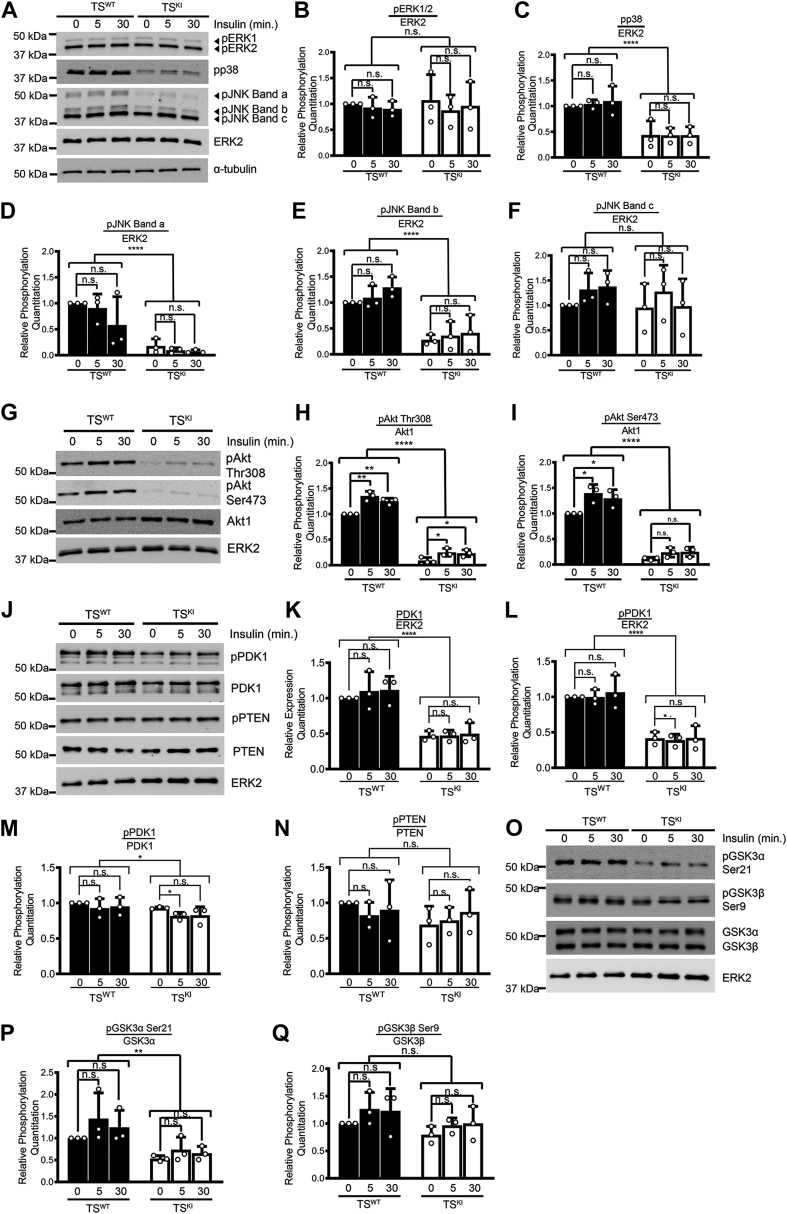
**MAP3K4 inactivation disrupts activation of specific pathways downstream of the IGF1R and IR.***A*–*F*, insulin does not stimulate MAPK signaling in TS cells. Phosphorylation of p38 and JNK is reduced in TS^KI^ cells relative to TS^WT^ cells regardless of insulin stimulation. Phosphorylated JNK bands are labeled bands a, b, and c. *B*–*F*, densitometry of Western blots detecting phosphorylation of (*B*) ERK1/2, (*C*) p38, and (*D–F*) JNK shows MAPK phosphorylation is not changed with insulin stimulation in TS cells. *B*, densitometry of phosphorylated ERK1 and ERK2 was combined into one measurement. *D*–*F*, phosphorylated JNK bands detected by Western blotting are labeled bands a, b, and c. *B*–*F*, expression was normalized to ERK2. *G*–*I*, reduced activation of Akt by insulin in TS^KI^ cells relative to TS^WT^ cells. *H* and *I*, densitometry analyses are shown with phosphorylation normalized to Akt1. *J*–*N*, reduced PDK1 expression and phosphorylation at Ser241 in TS^KI^ cells relative to TS^WT^ cells. *K*–*N*, densitometry analyses are shown. PDK1 expression was normalized to ERK2. PDK1 phosphorylation was normalized to ERK2 or PDK1. PTEN inhibitory phosphorylation at Ser380/Thr382/Thr383 was normalized to PTEN protein levels. *O*–*Q*, reduced phosphorylation of GSK3α in TS^KI^ cells relative to TS^WT^ cells. *P* and *Q*, densitometry analyses are shown. Phosphorylated GSK3α was normalized to total GSK3α protein, and phosphorylated GSK3β was normalized to total GSK3β protein. *A*, *G*, *J*, and *O*, Western blot images are representative of 3 biologically independent experiments. *J* and *O* show images from the same biological sample set and share the same ERK2 blot used as a loading control. *B*, *C*, *D*, *E*, *F*, *H*, *I*, *K*, *L*, *M*, *N*, *P*, and *Q*, densitometry was used to quantify 3 biologically independent experiments. Graphs show the mean ± SD. ∗*p* < 0.05; ∗∗*p* < 0.01; ∗∗∗∗*p* < 0.0001; Student’s *t* test; two-way ANOVA. Akt, protein kinase B; ERK1/2, extracellular-regulated kinase 1/2; GSK3α, glycogen synthase kinase 3 alpha; IGF1R, insulin-like growth factor 1 receptor; IR, insulin receptor; JNK, c-Jun N-terminal kinase; KI, kinase inactive; MAPK, mitogen-activated protein kinase; MAP3K4, mitogen-activated protein kinase kinase kinase 4; ns, not significant; PDK1, 3-phosphoinositide-dependent protein kinase 1; PTEN, phosphatase and tensin homolog; TS, trophoblast stem; Insulin, 25 μg/ml.

Stimulation of the IGF1R and IR promotes cellular growth through the MAPK and Akt signaling pathways in several cell types (22). ERK is activated mainly by growth-promoting signals and has previously been shown to be stimulated by insulin in multiple cell types, including adipocytes, skeletal muscle, vascular smooth muscle, and hepatocytes (44, 45, 46, 47). However, insulin stimulation had no significant effect on ERK phosphorylation in either TS^WT^ or TS^KI^ cells (Fig. 8, *A* and *B*). p38 and JNK are primarily activated by cellular stress but have also been shown to be stimulated by insulin in some cell types such as adipocytes and skeletal muscle (44, 45, 47). The JNK family is encoded by 3 genes: *Jnk1*, *Jnk2*, and *Jnk3* (48). However, based on RNA-sequencing data, only *Jnk1* and *Jnk2* are expressed in TS cells (17). Furthermore, *Jnk1* and *Jnk2* transcripts can each be spliced up to 4 different ways, resulting in multiple bands detected by Western blotting (48). Consistent with previous observations by Abell *et al.* (15), TS^KI^ cells exhibited reduced basal phosphorylation of p38 and JNK when compared with TS^WT^ cells (Fig. 8, *A* and *C–F*). Importantly, in the presence of insulin, phosphorylation of p38 and JNK remained reduced in TS^KI^ cells (Fig. 8, *A* and *C–F*). Furthermore, insulin stimulation had no significant effect on the phosphorylation of p38, JNK, or ERK in either TS^WT^ or TS^KI^ cells (Fig. 8, *A–F*). These data suggest that MAP3K4 promotes the activity of p38 and JNK. Furthermore, these data indicate that the MAPKs ERK, p38, and JNK are not stimulated by high levels of insulin in TS cells.

Insulin stimulation has been shown to activate the Akt signaling pathway in many cell types. Based on the reduced Akt signaling in TS^KI^ cells under basal conditions, we examined the impact of MAP3K4 inactivation on insulin stimulation of this pathway. Because of the requirement for serum to maintain stemness of TS cells, all insulin-stimulated experiments were performed in the presence of serum. Insulin treatment increased the phosphorylation of Akt at Thr308 and Ser473 in TS^WT^ cells when compared with control-treated TS^WT^ cells (Fig. 8, *G*–*I*). However, high-dose insulin treatment of TS^KI^ cells failed to induce Akt dual phosphorylation (Fig. 8, *G*–*I*). The absence of insulin-stimulated Akt dual phosphorylation in TS^KI^ cells was consistent with the lack of insulin-stimulated IGF1R and IR kinase domain phosphorylation, suggesting that the Akt pathway cannot be fully activated in TS^KI^ cells by means of IGF1R/IR stimulation (compare Figs. 7, *A–C* and 8, *G–I*). We also examined expression and phosphorylation of PDK1 and PTEN, upstream regulators of Akt. Consistent with examination under basal conditions (Fig. 4), total and phosphorylated levels of PDK1 were reduced in TS^KI^ cells relative to TS^WT^ cells regardless of the absence or the presence of insulin (Fig. 8, *J–M*). Furthermore, normalization of phosphorylated PDK1 to total PDK1 levels demonstrated that decreased PDK1 phosphorylation was due to reduced protein expression (Fig. 8, *J–M*). Insulin did not increase the phosphorylation of PDK1 at Ser241. Although phosphorylation of this site is essential for PDK1 activity, previous studies have shown that insulin stimulation does not increase the expression or phosphorylation of PDK1 (36). Total and phosphorylated PTEN levels were similar in unstimulated and insulin-stimulated TS^KI^ cells relative to TS^WT^ cells, suggesting PTEN is not altered in TS^KI^ cells (Fig. 8, *J* and *N*). Although phosphorylation of GSK3α Ser21 was decreased in all TS^KI^ cell samples, insulin did not result in statistically significant changes in GSK3 phosphorylation (Fig. 8, *O–Q*). This lack of responsiveness of GSK3 to insulin may be due to the presence of serum in the culture medium. Taken together, these data suggest that MAP3K4 promotes the Akt signaling pathway in TS cells but not the ERK pathway. Furthermore, the Akt pathway is the primary signaling pathway stimulated by insulin in TS cells and not the MAPK pathways. Significantly, ablation of MAP3K4 activity prevents Akt activation.

### Alterations in the IGF1R, IR, and Akt signaling pathway persist in differentiated cells formed by directed *in vitro* differentiation of MAP3K4 KI TS cells

**Figure 9:**
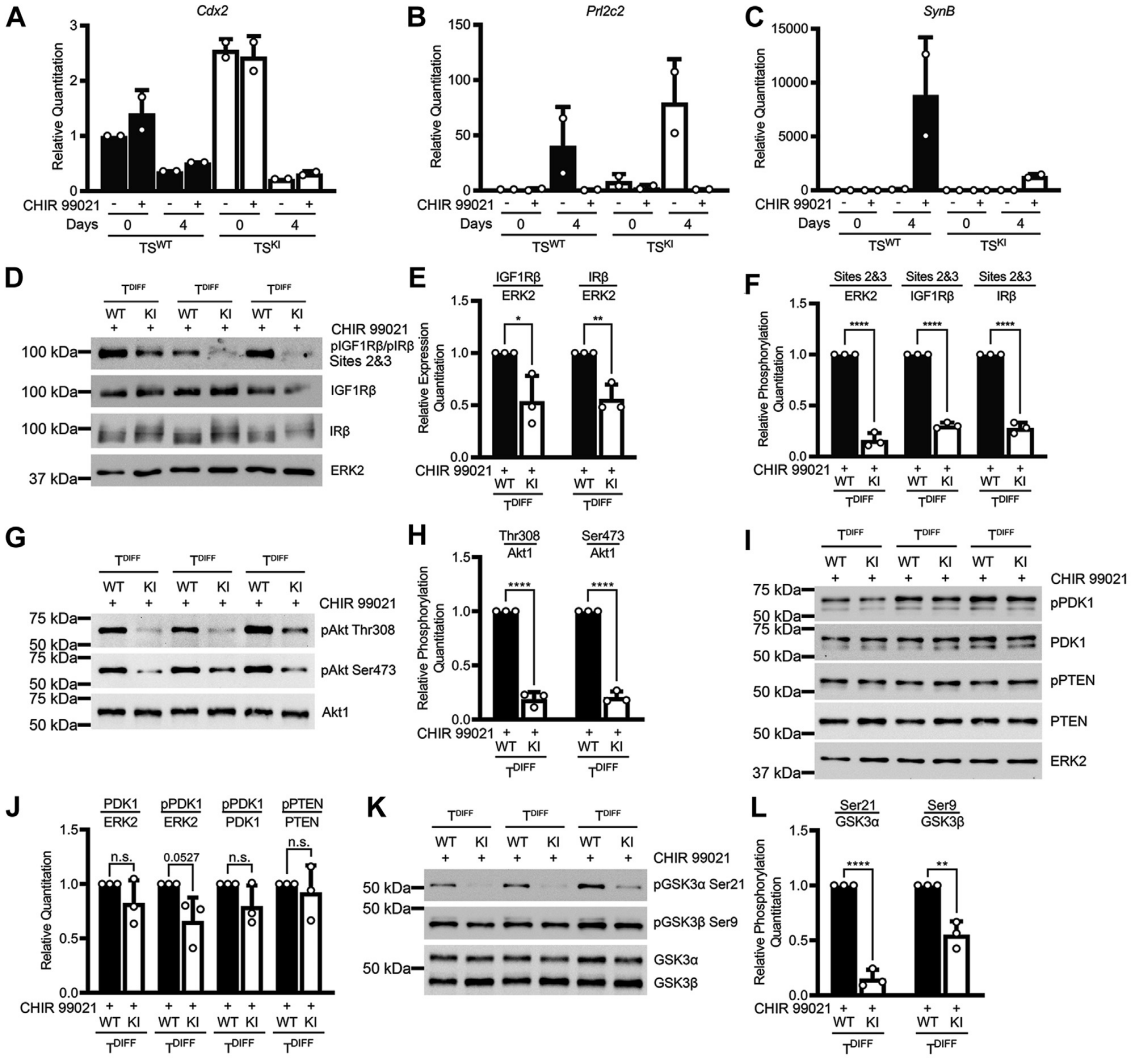
**Reduced IGF1R/IR and Akt signaling pathway in T**^**DIFF**^**cells generated by directed *in vitro* differentiation of MAP3K4 kinase–inactive TS cells to syncytiotrophoblasts.***A–C*, MAP3K4 inactivation disrupts the *in vitro* differentiation of TS cells to syncytiotrophoblasts. qPCR data normalized to *Gapdh* are expressed as a fold change relative to undifferentiated TS^WT^ cells and are the mean ± range of 2 biologically independent experiments. TS cells were either cultured under stem cell conditions (0) or differentiated for 4 days (4) in the absence or the presence of the GSK3 inhibitor CHIR 99021. (*Cdx2*, stem marker; *Prl2c2*, giant cell marker, *Synb*, SynT-II marker). *D*–*F*, reduced expression and phosphorylation of the IGF1R and IR after CHIR-directed TS cell differentiation to syncytiotrophoblasts (T^DIFF^). Site 2 represents pIGF1Rβ Tyr1135 and pIRβ Tyr1150, and site 3 represents pIGF1Rβ Tyr1136 and pIRβ Tyr1151. Western blotting images simultaneously show 3 biologically independent experiments. *E* and *F*, densitometry analyses are shown. Total IGF1Rβ and IRβ protein expression was normalized to ERK2. Phosphorylated IGF1Rβ and IRβ were normalized to ERK2, IGF1Rβ, or IRβ as indicated. *G* and *H*, reduced phosphorylation of Akt in KI trophoblasts after directed TS cell differentiation. *G*, Western blotting images simultaneously show 3 biologically independent experiments. *H*, densitometry analyses show normalization of phosphorylated Akt to total Akt1. *I* and *J*, PDK1 expression and Ser241 phosphorylation after directed TS cell differentiation. *I*, Western blotting images simultaneously show 3 biologically independent experiments. *J*, PDK1 expression was normalized to ERK2. PDK1 phosphorylation was normalized to ERK2 or PDK1. PTEN inhibitory phosphorylation at Ser380/Thr382/Thr383 was normalized to PTEN protein levels. *K* and *L*, reduced phosphorylation of GSK3α and GSK3β in KI cells after directed TS cell differentiation. *K*, Western blotting images simultaneously show 3 biologically independent experiments. *L*, densitometry analyses with phosphorylated GSK3α normalized to total GSK3α protein, and phosphorylated GSK3β normalized to total GSK3β protein. *E*, *F*, *H*, *J*, and *L*, densitometry analyses show the mean ± SD of 3 biologically independent experiments. ∗*p* < 0.05; ∗∗*p* < 0.01; ∗∗∗∗*p* < 0.0001; Student’s *t* test. Akt, protein kinase B; ERK2, extracellular-regulated kinase 2; GSK3, glycogen synthase kinase 3; IGF1R, insulin-like growth factor 1 receptor; IR, insulin receptor; KI, kinase inactive; MAP3K4, mitogen-activated protein kinase kinase kinase 4; ns, not significant; PDK1, 3-phosphoinositide-dependent protein kinase 1; PTEN, phosphatase and tensin homolog; qPCR, quantitative PCR; TS, trophoblast stem; CHIR 99021, 3 μM.

TS cell maintenance requires culture in the presence of FGF4 and MEF-CM, and removal results in differentiation of TS cells to a heterogeneous population of trophoblast subtypes of the placenta, including syncytiotrophoblasts, spongiotrophoblasts, and trophoblast giant cells (14, 15). With undirected TS cell differentiation induced simply by the removal of FGF4 and MEF-CM, TS cells preferentially differentiate *in vitro* to trophoblast giant cells with very few syncytiotrophoblasts formed (49). These results are in stark contrast to *in vivo* differentiation where cells of the labyrinth layer including syncytiotrophoblasts make up the largest layer of the placenta (12, 49). In the placenta, IGF1R is expressed in the labyrinth by a specific lineage of syncytiotrophoblasts called syncytiotrophoblast layer II (SynT-II) cells (50). Directed *in vitro* differentiation to SynT-II cells is induced by removal of FGF4 and MEF-CM in the presence of the GSK3 inhibitor CHIR 99021 (49). CHIR 99021 inhibits both GSK3α and GSK3β, resulting in reduced phosphorylation of tyrosine 279/216 (GSK3α/GSK3β) (51, 52). Removal of FGF4 and MEF-CM resulted in decreased expression of the TS cell stemness marker *Cdx2* in both TS^WT^ and TS^KI^ cells (Fig. 9*A*). These decreases occurred in the absence or the presence of GSK3 inhibitor (Fig. 9*A*). Differentiation in the absence of CHIR 99021 resulted in induction of trophoblast giant cells that express *Prl2c2* but very little expression of the SynT-II marker *SynB* (Fig. 9, *B* and *C*). In contrast, differentiation in the presence of the GSK3 inhibitor CHIR 99021 resulted in strong expression of *SynB* by differentiated WT cells (Fig. 9*C*). Interestingly, TS^KI^ cells differentiated in the presence of CHIR 99021 showed poor expression of *SynB*, suggesting that TS^KI^ cells were not differentiating to the SynT-II cells that are known to express IGF1R (Fig. 9*C*). The spongiotrophoblast marker *Tpbpa* was undetectable under all conditions, consistent with differentiation to this cell type requiring 6 to 10 days of *in vitro* differentiation in the absence of CHIR (data not shown) (17). Comparison of phosphorylated IGF1Rβ/IRβ levels after differentiation in the presence of CHIR 99021 in 3 independent experimental sample sets showed reduced phosphorylated IGF1Rβ/IRβ in differentiated KI cells compared with differentiated WT cells (Fig. 9, *D–F*). Reduced phosphorylation was observed regardless of normalization to ERK2, IGF1Rβ, or IRβ (Fig. 9*F*). Normalization of total IGF1Rβ and IRβ levels to ERK2 showed modest but statistically significant decreases in the expression of both receptors in differentiated KI cells compared with differentiated WT cells (Fig. 9*E*). Trophoblast cells derived from differentiation of TS^WT^ cells in the presence of CHIR 99021 showed robust phosphorylation of Akt at both Thr308 and Ser473 that was reduced in trophoblasts derived from differentiation of TS^KI^ cells in the presence of CHIR 99021 (Fig. 9, *G* and *H*). Although total and phosphorylated PTEN levels were similar, phosphorylated PDK1 and total PDK1 protein showed modest decreases in differentiated trophoblasts, but these did not reach statistical significance (Fig.9, *I* and *J*). Consistent with reduction of Akt phosphorylation in CHIR-treated KI cells compared with WT, phosphorylation of the Akt targets GSK3α and GSK3β were all decreased (Fig. 9, *K* and *L*). Together, these data show that TS^KI^ cells have impaired *in vitro* differentiation to SynT-II cells, suggesting a role for MAP3K4 in differentiation to this cell type. Furthermore, these data support a potential role for MAP3K4 regulation of the IGF1R/IR and Akt signaling pathway during differentiation to SynT-II cells.

### Alterations in the IGF1R/IR and Akt pathway persist *in vivo* in differentiated placental tissues formed by MAP3K4 KI TS cells

**Figure 10:**
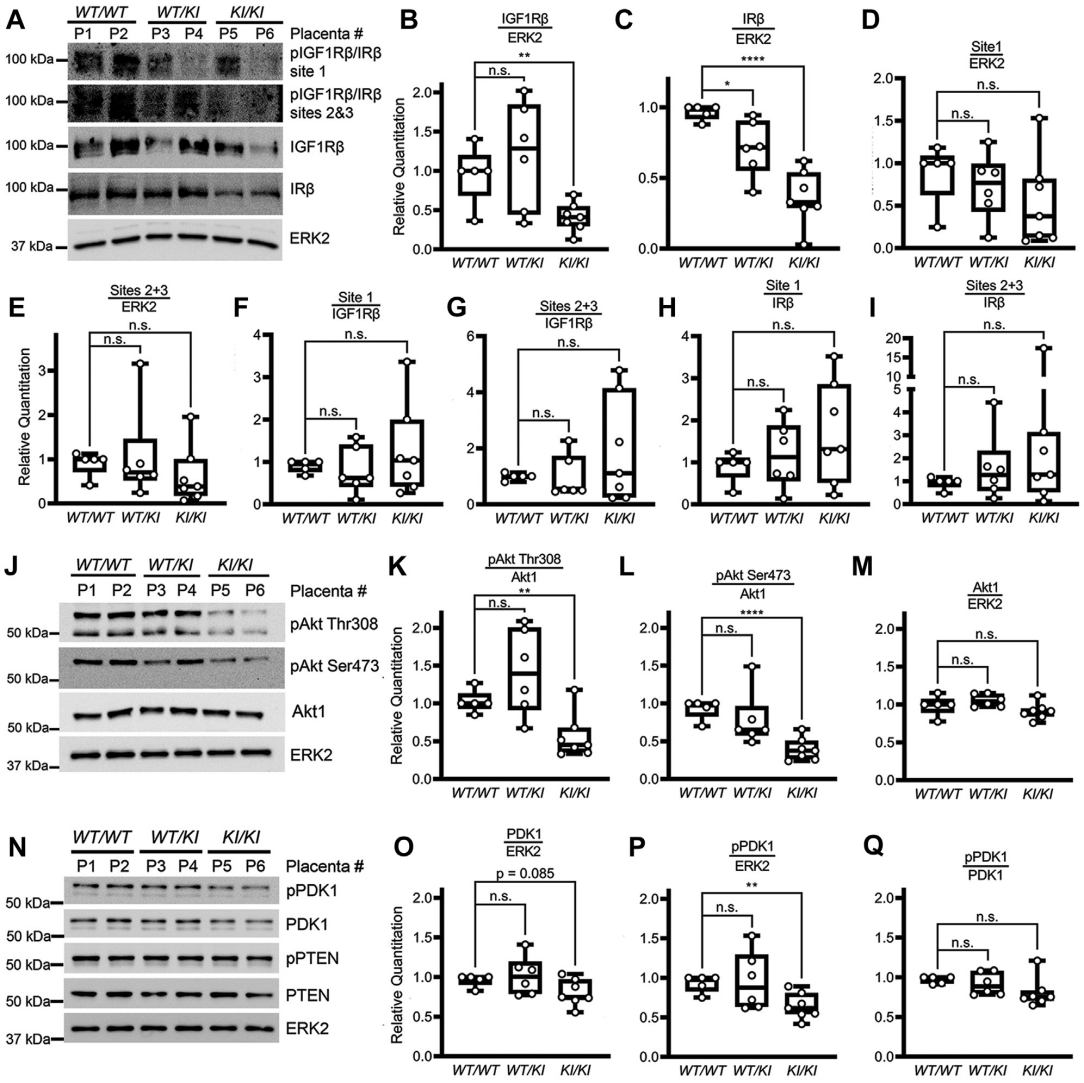
**The IGF1R/IR and Akt pathway is inhibited *in vivo* in the placentas of *Map3k4***^***KI/KI***^**individuals.***A*, IGF1Rβ and IRβ expression is reduced in *Map3k4*^*KI/KI*^ E13.5 placentas. *B* and *C*, IGF1Rβ and IRβ expression normalized to ERK2. *D* and *E*, IGF1Rβ and IRβ phosphorylation normalized to ERK2. *F* and *G*, IGF1Rβ and IRβ phosphorylation normalized to IGF1Rβ. *H* and *I*, IGF1Rβ and IRβ phosphorylation normalized to IRβ. *J*–*M*, reduced Akt phosphorylation in *Map3k4*^*KI/KI*^ E13.5 placentas. Densitometry analyses with (*K, L*) Akt phosphorylation normalized to Akt1 and (*M*) Akt1 expression normalized to ERK2. *N*–*Q*, PDK1 expression and phosphorylation are reduced *Map3k4*^*KI/KI*^ E13.5 placentas. Densitometry analyses with PDK1 expression normalized to ERK2 (*O*), PDK1 phosphorylation normalized to ERK2 (*P*), or to PDK1 (*Q*). *A*, *J*, and *N*, Western blotting images show 2 independent placentas for each genotype from 1 litter and are representative of 18 independent placental extractions. *B*–*I*, *K*–*M*, and *O–Q*, densitometry was used to quantify 18 independent placenta extractions. Data are displayed as *box plots*; each point represents one individual placenta. ∗*p* < 0.05; ∗∗*p* < 0.01; ∗∗∗∗*p* < 0.0001; Student’s *t* test. Akt, protein kinase B; ERK2, extracellular-regulated kinase 2; IGF1R, insulin-like growth factor 1 receptor; IR, insulin receptor; KI, kinase inactive; *Map3k4*, mitogen-activated protein kinase kinase kinase 4; ns, not significant.

Placental tissue includes trophoblast subtypes derived from TS cell differentiation, endothelial cells derived from the fetal mesenchyme, and decidual cells derived from the mother (12). To determine if alterations in the IGF1R, IR, and Akt pathway seen in the TS^KI^ cells also occurs in TS cell–derived tissues, we isolated E13.5 placentas from *Map3k4*^*WT/KI*^ crosses in the 129/SvEv background and examined the expression and phosphorylation of the IGF1R, IR, and Akt pathway. *Map3k4*^*KI/KI*^ placentas showed statistically significant reductions in total IGF1Rβ and IRβ levels when compared with *Map3k4*^*WT/WT*^ placentas, suggesting a loss of IGF1R and IR expression in *Map3k4*^*KI/KI*^ placentas (Fig. 10, *A–C*). Phosphorylation of IGF1Rβ and IRβ normalized to total ERK2 showed decreases in *Map3k4*^*KI/KI*^ placentas relative to *Map3k4*^*WT/WT*^ placentas; however, these changes did not reach statistical significance (Fig. 10, *A* and *D*, and *E*). When IGF1Rβ/IRβ phosphorylation was normalized to either total IGF1Rβ or IRβ protein, there were no significant differences in phosphorylation, suggesting that reductions of active IGF1R and IR were due to reduced expression of these proteins (Fig. 10*A*, *F–I*). Taken together, these data suggest that MAP3K4 promotes the expression of the IGF1R and IR in cells differentiated *in vivo* in the placenta, and decreased expression of these receptors with loss of MAP3K4 activity results in reduced levels of IGF1R/IR activity.

### Reduced *in vivo* Akt phosphorylation in MAP3K4 KI placentas

Specific MAPKs are essential for TS cell maintenance, differentiation, and placental development (53, 54, 55, 56). FGF4 stimulates phosphorylation of ERK, JNK, and p38 in TS cells. Differentiation of TS cells by removal of FGF4 induces the loss of phosphorylation of ERK, JNK, and p38 (15). p38α knockout mice display embryonic lethality at E11.5 because of insufficient oxygen and nutrient transfer of the placenta. ERK2 deficiency also results in embryonic lethality at E11.5 because of poor placental vascularization and reduced labyrinth size (54, 56). Abell *et al.* (15) reported that TS^KI^ cells have reduced FGF4-stimulated phosphorylation of p38 and JNK. However, FGF4-stimulated ERK phosphorylation was similar between TS^WT^ and TS^KI^ cells (15). Upon removal of FGF4 and MEF-CM, differentiation-induced reduction of JNK and p38 phosphorylation occurred earlier in TS^KI^ cells relative to TS^WT^ cells (15). After *in vitro* differentiation, phosphorylation of ERK, JNK, and p38 was similar between WT and KI cells (15). However, phosphorylation of MAPKs in *Map3k4*^*KI/KI*^ placentas was not previously examined. Consistent with previously published *in vitro* experiments with TS cells, ERK phosphorylation was similar between *Map3k4*^*WT/WT*^ and *Map3k4*^*KI/KI*^ 129/SvEv placentas (Fig. S5, *A–B*) (15). JNK phosphorylation was also similar between *Map3k4*^*WT/WT*^ and *Map3k4*^*KI/KI*^ placentas (Fig. S5, *A–D*). However, phosphorylation of p38 was difficult to detect in the E13.5 placenta, preventing the assessment of this MAPK (data not shown). These findings in the placenta were consistent with the lack of differences in phosphorylation of MAPK proteins in the *in vitro* differentiated WT and KI cells (15).

Akt is expressed throughout the placenta, and genetic deletion of Akt1 results in fetal and placental growth deficiencies (57). Furthermore, Akt1 null mice exhibit reduced bodyweight and delayed growth similar to *Map3k4*^*KI/KI*^ mice (58). Based on the reduced placental area and weight in *Map3k4*^*KI/KI*^ placentas when compared with *Map3k4*^*WT/WT*^ placentas and the loss of Akt phosphorylation in TS^KI^ cells when compared with TS^WT^ cells, we predicted that phosphorylation of Akt may be diminished in *Map3k4*^*KI/KI*^ placentas. Akt phosphorylation at both Thr308 and Ser473 was significantly reduced in *Map3k4*^*KI/KI*^ 129/SvEv placentas when compared with *Map3k4*^*WT/WT*^ placentas, suggesting that Akt activity is reduced in *Map3k4*^*KI/KI*^ placentas (Fig. 10, *J–L*). Total Akt1 protein levels were similar among all genotypes (Fig. 10*M*). When normalized to total ERK2, phospho and total protein levels of PDK1 were also decreased in *Map3k4*^*KI/KI*^ placentas; however, normalization of phospho-PDK1 to total PDK1 protein demonstrated that decreases in phospho-PDK1 resulted from reduced total PDK1 protein expression (Fig. 10, *N–Q*). Total and phosphorylated PTEN levels were not affected by *Map3k4* genotype (Fig. 10*N* and S5, *E–G*). GSK3α/β phosphorylation was not significantly altered in the placenta (Fig. S5, *H–J*). Taken together, these data show that the Akt signaling pathway is disrupted in MAP3K4 KI placentas, indicating a possible cause for placental insufficiency and FGR in KI individuals.

### MAP3K4 promotes the IGF1R/IR and Akt signaling pathway by activation of CBP and repression of HDAC6

**Figure 11:**
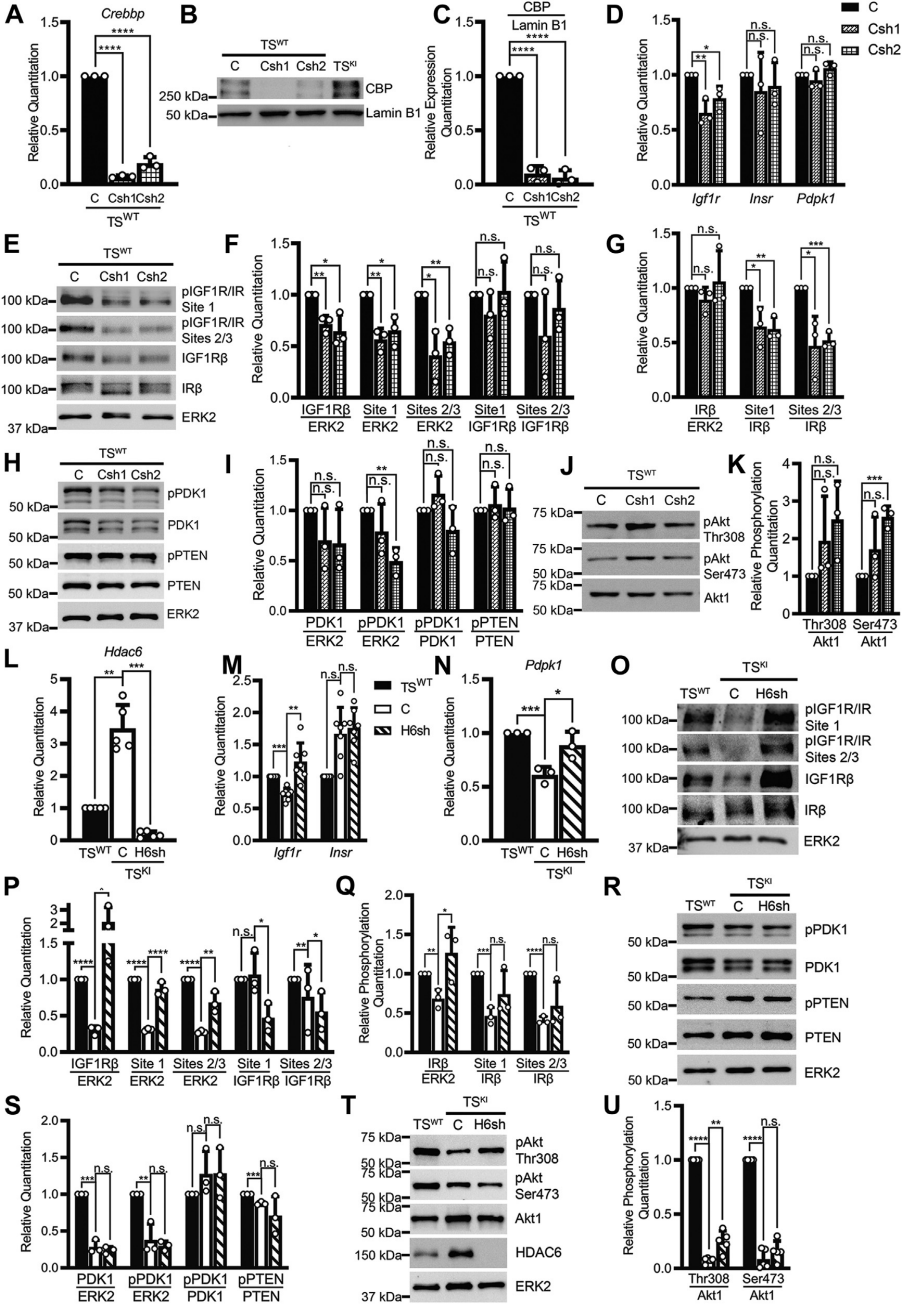
**Coregulation of *Igf1r* expression by MAP3K4, CBP, and HDAC6.** CBP promotes *Igf1r* expression, and HDAC6 represses *Igf1r* and *Pdpk1* expression. *A*, shRNA knockdown of *Crebbp* in TS^WT^ cells using 2 independent shRNAs. *Crebbp* transcript expression measured using quantitative PCR (qPCR) in 3 biologically independent experiments. *B* and *C*, shRNA knockdown of *Crebbp* in TS^WT^ cells reduces CBP protein expression as measured by (*B*) Western blotting of nuclear lysates and (*C*) densitometry analyses normalized to lamin B1. *D*, shRNA knockdown of *Crebbp* results in decreased *Igf1r* transcript expression as measured by qPCR in 3 biologically independent experiments. *E*–*G*, knockdown of CBP results in decreased expression and phosphorylation of IGF1Rβ. *F* and *G*, densitometry analyses with normalization as indicated. *H*–*K*, knockdown of CBP on PDK1, PTEN, and AKT expression and phosphorylation. *I*, densitometry analyses with normalization as indicated. *K*, densitometry analyses with normalization of phosphorylated Akt to Akt1. *L*, MAP3K4 kinase inactivation in TS cells results in increased *Hdac6* expression that is reduced by *Hdac6* shRNA knockdown (H6sh) in TS^KI^ cells. qPCR data show five biologically independent experiments. *M, Hdac6* shRNA knockdown increases *Igf1r* transcript expression. qPCR data show 7 biologically independent experiments. *N, Hdac6* shRNA knockdown increases *Pdpk1* transcript expression. qPCR data show 3 biologically independent experiments. *O–Q*, reduction of HDAC6 expression in TS^KI^ cells through *Hdac6* shRNA knockdown restores IGF1Rβ/IRβ expression and phosphorylation. *P* and *Q*, densitometry analyses with normalization as indicated. *R*–*U*, impact of *Hdac6* shRNA knockdown on PDK1, PTEN, and Akt expression and phosphorylation. *S*, densitometry analyses with normalization of phosphorylated or total protein as indicated. *U*, densitometry analyses with normalization of phosphorylated Akt to Akt1. Data show five biologically independent experiments. *A*, *D*, *L*–*N*, qPCR data normalized to *Rps11* are expressed as a fold change relative to TS^WT^ cells and show the mean ± SD. *B*, *E*, *H*, *J*, *O*, and *R*, Western blotting images are representative of 3 biologically independent experiments. *T*, Western blotting images are representative of five biologically independent experiments. *C*, *F*, *G*, *I*, *K*, *P*, *Q*, and *S*, densitometry was used to quantify 3 biologically independent experiments. *U*, densitometry was used to quantify five biologically independent experiments. ∗*p* < 0.05; ∗∗*p* < 0.01; ∗∗∗*p* < 0.001; ∗∗∗∗*p* < 0.0001; Student’s *t* test. AKT, protein kinase B; CBP, CREB-binding protein; HDAC6, histone deacetylase 6; MAP3K4, mitogen-activated protein kinase kinase kinase 4; ns, not significant; PDK1, 3-phosphoinositide-dependent protein kinase 1; PTEN, phosphatase and tensin homolog; TS, trophoblast stem.

A key question remaining related to the mechanism(s) by which MAP3K4 promotes the IGF1R/IR/Akt pathways and how the absence of MAP3K4 kinase activity disrupts this pathway. We clearly demonstrated that for the IGF1R and PDK1 components of the pathway, *Igf1r* and *Pdpk1* transcript levels were reduced with MAP3K4 inactivation (Figs. 3*G* and 4*G*). We have previously demonstrated that MAP3K4 activates the histone acetyltransferase CBP but does not control CBP expression (17). In addition, MAP3K4 inhibits the expression and activity of the histone deacetylase HDAC6 (59). Inactivation of MAP3K4 activity results in loss of CBP activity and increased expression and activity of HDAC6 (17, 59). These changes in these key epigenetic regulators resulted in reduced histone acetylation on the promoters of many genes related to maintenance of the epithelial state (17, 59, 60). Based on these previous studies, we predicted that CBP and/or HDAC6 may control *Igf1r* and *Pdpk1* expression. Expression of 2 independent *Crebbp* shRNAs in TS^WT^ cells resulted in reduced *Crebbp* transcript and CBP protein (Fig. 11, *A–C*). shRNA knockdown of CBP resulted in statistically significant decreases in *Igf1r* transcript (Fig. 11*D*). In contrast, *Insr* and *Pdpk1* transcript levels were unchanged by CBP knockdown (Fig. 11*D*). Reductions in *Igf1r* transcript were translated into reduced IGF1Rβ protein as measured by Western blotting, whereas IRβ protein levels were unchanged (Fig. 11, *E–G*). Loss of CBP resulted in reduced phosphorylation of the IGF1Rβ and IRβ; however, densitometry analyses revealed that reduced phosphorylation was due to reduced IGF1Rβ protein expression (Fig. 11, *E–G*). Phosphorylation of PDK1 was also decreased with CBP knockdown (Fig. 11, *H* and *I*). Densitometry analyses showed that decreases in PDK1 phosphorylation were due to decreased PDK1 expression (Fig. 11, *H* and *I*). However, these changes in PDK1 did not result in the reduction of Akt phosphorylation (Fig. 11, *J* and *K*). Together, these data suggested that MAP3K4 promotes the IGF1R/IR pathway by stimulating CBP-mediated expression of the *Igf1r*.

**Figure 12:**
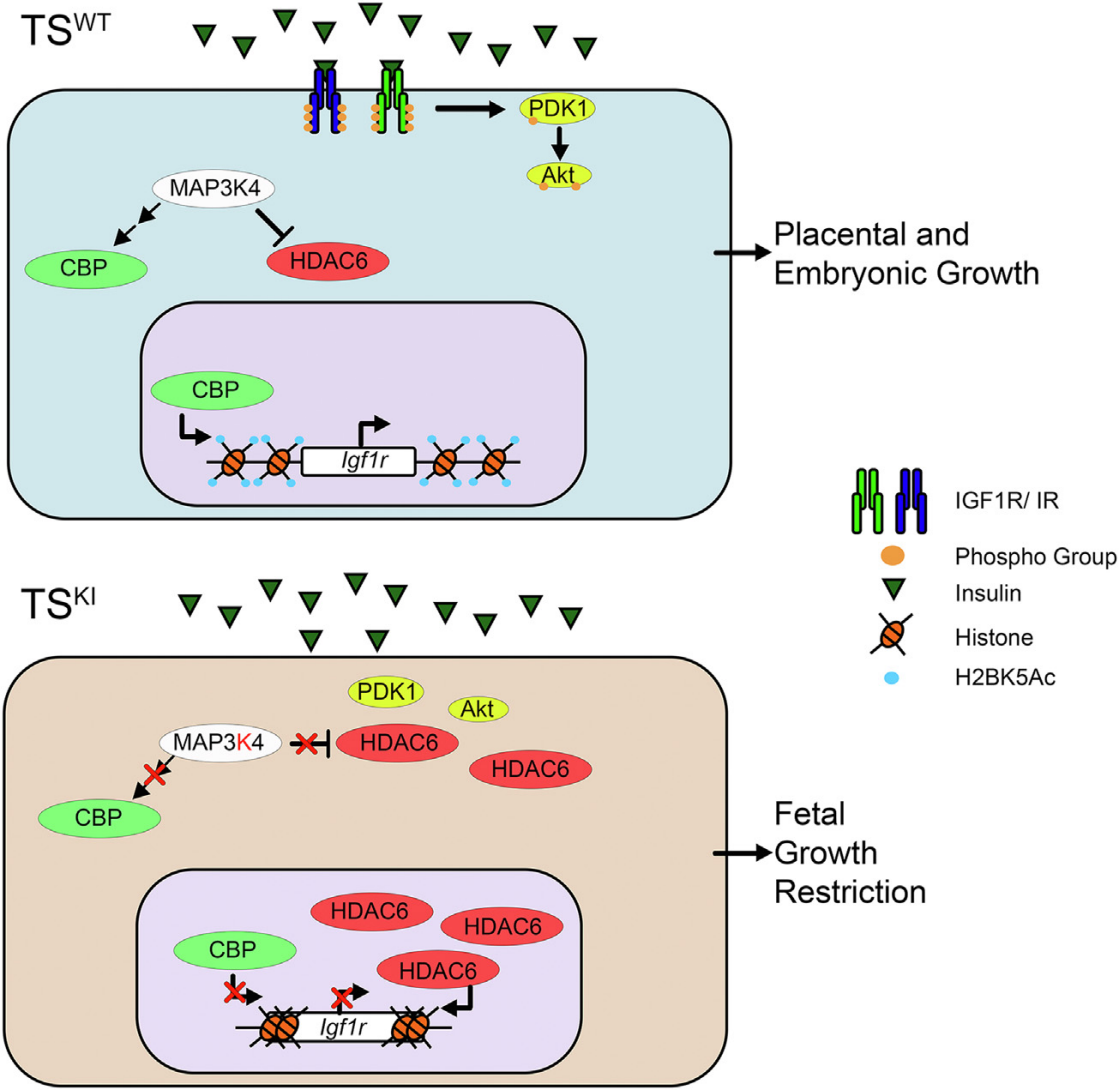
**MAP3K4 regulates the expression and phosphorylation of key components of the IGF1R signaling axis by controlling the expression of *Igf1r*.** MAP3K4 promotes *Igf1r* transcript expression through CBP activation in TS^WT^ cells. Localized to the cell surface, IGF1R is autophosphorylated upon ligand stimulation. Activation of the receptor induces the phosphorylation of downstream signaling components PDK1 and Akt, promoting placental and embryonic growth. In TS^KI^ cells, *Igf1r* transcript is repressed because of reduced CBP activity and increased HDAC6 expression and activity. Reduced IGF1R expression from MAP3K4 kinase inactivation impairs IGF1R activation of the Akt pathway. Disruption of the IGF1R/IR and Akt signaling pathway because of MAP3K4 kinase inactivation results in placental insufficiency and FGR. Akt, protein kinase B; CBP, CREB-binding protein; FGR, fetal growth restriction; HDAC6, histone deacetylase 6; IGF1R, insulin-like growth factor 1 receptor; MAP3K4, mitogen-activated protein kinase kinase kinase 4; PDK1, 3-phosphoinositide-dependent protein kinase 1; TS, trophoblast stem.

Based on our previous studies demonstrating the key role of MAP3K4 in repressing HDAC6 expression and activity, we also examined the impact of HDAC6 on the components of the IGF1R/IR and Akt pathway (17, 59). *Hdac6* is overexpressed in TS^KI^ cells compared with TS^WT^ cells, and shRNA knockdown is sufficient to reduce *Hdac6* transcript to low levels (Fig. 11*L*). We were only able to identify 1 shRNA capable of reducing *Hdac6* expression (59). shRNA knockdown of *Hdac6* in TS^KI^ cells was sufficient to restore the transcript levels of both *Igf1r* and *Pdpk1* (Fig. 11, *M* and *N*). However, *Hdac6* knockdown did not result in statistically significant changes in *Insr* transcript (Fig. 11*M*). Interestingly, loss of HDAC6 robustly increased the protein expression of both IGF1Rβ and IRβ protein (Fig. 11, *O–Q*). Phosphorylation of all 3 sites on IGF1Rβ/IRβ was also restored by *Hdac6* knockdown in TS^KI^ cells to levels observed in TS^WT^ cells (Fig. 11, *O–Q*). However, comparison of normalization of changes in IGF1Rβ/IRβ phosphorylation to either total ERK2, IGF1Rβ, or IRβ protein expression revealed that increases in phosphorylation were due to increased protein expression of the IGF1Rβ and IRβ with *Hdac6* knockdown (Fig. 11, *O–Q*). Although HDAC6 repressed *Pdpk1* transcript expression, shRNA knockdown of *Hdac6* in TS^KI^ cells failed to restore PDK1 protein expression or phosphorylation (Fig. 11, *R* and *S*). *Hdac6* shRNA knockdown in TS^KI^ cells resulted in modest but statistically significant increases in phosphorylation of Akt at Thr308 but no change in Ser473 (Fig. 11, *T* and *U*). These weak effects of *Hdac6* shRNA knockdown on PDK1 and Akt phosphorylation may be due to the well-defined role of the HDAC6 target heat shock protein 90 in the folding, stability, and signaling of these proteins (61, 62). These data suggest that HDAC6 mainly controls the IGF1R/IR/Akt pathway by inhibiting *Igf1r* and *Pdpk1* expression. However, HDAC6 effects on IRβ protein levels suggest that HDAC6 likely controls this pathway through additional mechanism(s). Altogether, these findings suggest that MAP3K4 promotes fetal and placental growth through control of CBP and HDAC6 regulation of *Igf1r* and *Pdpk1* expression (Fig. 12).

## Discussion

Herein, our findings demonstrate that MAP3K4 promotes embryonic and placental growth and survival. MAP3K4 KI causes highly penetrant lethality prior to weaning and restricted growth of adults. Loss of MAP3K4 kinase activity results in FGR in part because of placental insufficiency. We define a molecular mechanism by which MAP3K4 regulates the activity of the IGF1R, IR, and Akt signaling pathway primarily through control of the expression of the IGF1R. Importantly, we demonstrate that this mechanism occurs both *in vitro* in TS cells that give rise to trophoblasts and *in vivo* in the placenta. Overall, these data demonstrate a critical role for MAP3K4 in promoting fetal and placental growth by controlling the IGF1R/IR and Akt signaling pathway through regulation of IGF1R expression.

We have discovered a model of FGR caused by MAP3K4 KI. Importantly, MAP3K4 KI–induced FGR is observed in both the pure 129/SvEv and mixed 129/SvEv/C57BL/6N backgrounds. We predict that FGR will also occur in *Map3k4*^*KI/KI*^ mice in the pure C57BL/6N background. FGR-complicated pregnancies are a major public health concern because of increased perinatal mortality and morbidity and long-term consequences of FGR (2, 8, 9, 10). Unfortunately, therapies to prevent or treat FGR remain difficult to identify. Although some treatments that increase blood flow such as aspirin and heparin initially demonstrated a reduction of FGR, meta-analyses showed no reduced risk (63, 64). Possible interventions of FGR, including the direct introduction of growth factors to the mother and fetus, were tested in various animal models (65). These studies show a significant increase in fetal growth; however, long-term studies on the health of the mother and fetus have yet to be performed, and no clinical trials are currently underway (65). Effectiveness of growth factor treatment requires the correct expression and localization of the necessary receptors. Growth factor therapy would be predicted to be ineffective in conditions of FGR that have reduced expression of these receptors such as MAP3K4 KI. Reduced activation of IGF1R/IR because of MAP3K4 KI could be an unknown cause for FGR, highlighting the importance of a clearer understanding of the molecular mechanisms underlying FGR and more diverse models of FGR.

MAP3K4 KI leads to placental insufficiency and FGR. Precise regulation and timing of differentiation and epithelial-to-mesenchymal transition (EMT) are critical for normal placentation (17, 66, 67, 68). TS^KI^ cells exhibit a more mesenchymal phenotype including front-to-back polarity, increased expression of EMT markers, and increased invasiveness when compared with the epithelial TS^WT^ cells (17). One of the first developmental EMTs is the transition of the epithelial TE to more mesenchymal cells, which invade the uterine epithelium (67). This EMT event is essential for placentation and pregnancy (68). Following implantation, TE cells proliferate in response to FGF4 stimulation and form the stem cell compartment of the extraembryonic ectoderm. Eventually, extraembryonic ectoderm cells differentiate into invasive secondary trophoblast giant cells and displace the endothelial cell lining of maternal blood vessels. This process is critical for establishing blood flow to the placenta and maintaining pregnancy (12). Our work clearly shows a reduction of IGF1R/IR expression and phosphorylation in the cultured TS^KI^ cells. Furthermore, directed differentiation of TS^KI^ cells *in vitro* to IGF1R-expressing SynT-II cells of the labyrinth revealed reduced IGF1R/IR phosphorylation and reduced activation of the Akt pathway compared with differentiated WT cells. These findings were also observed with *in vivo* differentiation in the E13.5 placenta with significantly decreased IGF1R and IR expression and reduced Akt and PDK1 phosphorylation in *Map3k4*^*KI/KI*^ placentas. Although examination of the median IGF1R/IR phosphorylation showed a clear trend with decreasing phosphorylation in *Map3k4*^*KI/KI*^ placentas, these changes in placental IGF1R/IR phosphorylation did not reach statistical significance. These findings may reflect the heterogenous cell types, including different types of mature trophoblasts, cells derived from the fetal mesenchyme, and maternally derived cells that are present in these placental extracts. Furthermore, additional contributing factors such as maternally produced hormones and the uterine environment may impact differentiation *in vivo* (12). These factors may be responsible for the modest differences in IGF1R/IR phosphorylation between *in vitro* and *in vivo*–differentiated KI cells.

Herein, we show that MAP3K4 controls the transcript expression of *Igf1r* (Fig. 12). Inactivation of MAP3K4 kinase activity resulted in reduced *Igf1r* transcript levels. Disruption of CBP expression in TS^WT^ cells caused a significant decrease in *Igf1r* transcript, and reduction of HDAC6 levels in TS^KI^ cells restored expression of *Igf1r*. Examination of our previously published sequencing data supported our new findings that *Igf1r* is coregulated in TS cells by MAP3K4, CBP, and HDAC6 in an H2BK5Ac-dependent manner (60). Previous work showed that inactivation of MAP3K4 kinase activity results in reduced JNK-dependent activation of CBP and increased abundance of HDAC6 by disrupting its ubiquitination and destruction in TS cells (17, 59). Together, our studies suggest a pathway by which MAP3K4 directly promotes *Igf1r* transcript levels through activation of CBP and inhibition of HDAC6 (Fig. 12). Interestingly, MAP3K4 and HDAC6, but not CBP, controlled the transcript expression of *Pdpk1*, another key regulator of fetal and placental growth. We predict that MAP3K4 coregulation of both these genes enables MAP3K4 greater control over growth.

Although MAP3K4 inactivation controlled *Igf1r* transcript but not *Insr* transcript, MAP3K4 KI results in reduced total and phosphorylated IGF1R and IR. Signaling by the IGF1R and IR is also controlled through changes in localization and by post-translational modifications (69, 70). Protein glycosylation is an important post-translational modification that promotes protein folding and localization. The cell–cell adhesion protein epithelial cadherin (E-cadherin) requires O-glycosylation for localization to the cell surface of TS cells (71). Importantly, IGF1R colocalizes with E-cadherin in the TE, and disruption of this interaction results in reduced IGF1R phosphorylation and increased apoptosis of the TE (70). MAP3K4 and HDAC6 regulate the expression of O-GalNAc glycosyltransferase 3 (*Galnt3*), which is necessary for E-cadherin O-glycosylation and localization in TS cells (71). TS^KI^ cells have reduced expression of *Galnt3* and decreased O-GalNAc glycosylation of E-cadherin. shRNA knockdown of *Hdac6* in TS^KI^ cells restores *Galnt3* expression and E-cadherin glycosylation and localization (71). Significantly, *Hdac6* knockdown restored the expression and phosphorylation of both the IGF1R and IR. Based on computational predictions of O-glycosylation sites and protein structure, IGF1R and IR are predicted to have O-glycosylation sites, implicating GALNT3-dependent modifications of IGF/IRs as a potential regulator of IGF/insulin signaling (72). IGF1R may also be mislocalized in TS^KI^ cells because of improper localization of E-cadherin and/or lack of O-glycosylation of IGF1R/IR resulting in reduced receptor phosphorylation and activation. Unfortunately, using commercially available antibodies, we were unable to detect cell surface IGF1R or IR in TS cells by immunofluorescence assays, limiting our ability to investigate IGF1R and IR localization (data not shown). Future studies will attempt to elucidate additional mechanisms by which MAP3K4 regulates IGF1R and IR expression and activity.

*Map3k4*^*KI/KI*^ mice show extremely similar phenotypes to mice with disruptions in the IGF/insulin signaling pathway. IGF/insulin signaling is indispensable for fetal growth, and well-characterized models of FGR include manipulation of IGF/insulin signaling in mice (73). Tissue-specific *Igf2* knockout in the TE results in reductions in placental weights similar to *Map3k4*^*KI/KI*^ placentas (≈21% of WT placentas) (74). Homozygote *Igf1* null mice (*Igf1*^*−/−*^) display a 60% reduction in birth weight when compared with WT littermates (*Igf1*^*+/+*^) and perinatal lethality (75). Surviving *Igf1*^*−/−*^ mice exhibit a non-Mendelian ratio similar to *Map3K4*^*KI/KI*^ animals (≈5%) and were 45% the size of *Igf1*^*+/+*^ mice (75). *Igf1r* knockout mutants die immediately after birth from respiratory failure with birth weights at 45% of normal, and double knockout embryos of *Igf1r* and *Insr* exhibit a 70% reduction in fetal weight when compared with WT littermates (76). Similar to *Map3k4*^*KI/KI*^ 129/SvEv/C57BL/6N mixed background mice, *Insr* and *Igfr1* double knockout mutants also display male to female sex reversal (18, 77, 78). These overlapping phenotypes suggest that reduced activity of the IGF1R and IR may contribute to MAP3K4-dependent male to female sex reversal. The IGF1R/IR activate the downstream PI3K, PDK1, and Akt pathway. Importantly, phenotypes seen with manipulation of this pathway are strikingly similar to the *Map3k4*^*KI/KI*^ phenotype (79, 80, 81, 82). For example, previous reports show *Akt1* knockout (*Akt1*^−/−^) embryos and placentas are significantly smaller than WT littermates, and surviving mice remain small into adulthood (57, 58, 83, 84). *Map3k4*^*KI/KI*^ placentas and TS cells show reduced phosphorylation of Akt, but total Akt1 expression is similar, suggesting that reduced placental weight in *Map3k4*^*KI/KI*^ placentas is partially dependent on Akt activity.

In summary, our studies provide insights into the function of MAP3K4 in placental development and fetal growth. Based on our results, we propose that MAP3K4 plays a critical role in the IGF1R/IR and Akt signaling pathway in TS cells and placenta, and MAP3K4 KI results in FGR caused by placental insufficiency. Our work demonstrates the significance of MAP3K4 in the developing placenta and postnatal growth. Future studies will further examine the molecular mechanisms of MAP3K4-dependent regulation of the IGF1R, IR, and Akt pathway and elucidate possible interactions with MAP3K4-dependent proteins. Furthermore, MAP3K4 KI may also provide a greater understanding of human FGR and serve as a model for future therapeutic interventions.

## Experimental procedures

### Mouse models and dissections

MAP3K4 KI (*Map3K4*^*KI/KI*^) mice were generated as previously described (16). *Map3k4*^*KI/KI*^ mice were in either a mixed 129/SvEv/C57BL/6N background backcrossed 1 time with C57BL/6N, in a pure 129/SvEv background, or in an F5 C57BL/6N background. All experimental samples isolated were performed in these backgrounds as specified. Mice were maintained in each background through *Map3K4*^*WT/KI*^ crosses because of the lethality of the mutation. Mice generated were genotyped for the KI mutation. For examination of E13.5 embryos, placentas, and livers, timed matings of *Map3k4*^*WT/KI*^ mice were performed. Mice were checked each morning for a copulatory plug, and noon of the day detected was considered E0.5. Pregnant females were euthanized at E13.5, and placentas, embryos, and livers were isolated. Imaging of isolations was performed using a Leica M125C microscope and LASV4.12 software (Leica Microsystems). Embryos were euthanized by decapitation. Embryo length, placental area, and liver area were calculated in Adobe Photoshop.

### Ethics statement

All experiments using animals were approved by the Institutional Animal Care and Use Committee at the University of Memphis. All experiments using animals were performed according to institutional and National Institutes of Health guidelines and regulations.

### Cell lines and culture conditions

TS^WT^ and TS^KI^ cells were isolated as previously described from WT mice and mice with a MAP3K4 KI mutation (15). TS cells were cultured in 30% TS media (RPMI 1640, 20% heat-inactivated fetal bovine serum, 1% penicillin and streptomycin, 1% l-glutamine, 1% sodium pyruvate, and 100 μM β-mercaptoethanol) as well as 70% MEF-CM at 37 °C and 7% CO_2_. Complete TS media were supplemented with FGF4 (37.5 ng/ml) and heparin (1 μg/ml) for maintenance of stemness. For differentiation experiments, TS cells were initially seeded in complete TS media. The next day, cells were cultured in the absence of MEF-CM, FGF4, and heparin. Cells were treated daily with either vehicle control dimethyl sulfoxide (DMSO) or 3 μM CHIR 99021 (Selleck Chemical). For insulin treatment, cells were seeded in the previously described complete TS media for 4 days. On the day of harvest, cells were treated with either 25 μg/ml of insulin (Gibco; catalog no.: 12585-014) or phosphate-buffered saline for either 5 or 30 min prior to harvest. For experiments to inhibit ERBB2, cells were treated for 48 h with 1 μM lapatinib (MedChemExpress). For chronic inhibition of FGFR4, cells were treated for 48 h with either vehicle control (DMSO), 0.1 μM, or 1 μM BLU9931 (MedChemExpress). For chronic lapatinib and BLU9931 experiments, cells were treated every 24 h for 48 h. For acute BLU9931 experiments, cells were treated for 1 h with either vehicle control (DMSO) or 1 μM BLU9931. shRNA knockdown using lentiviral shRNAs in TS cells was performed as described previously (17). Cell lines with *Crebbp* knockdown or *Hdac6* knockdown were created as previously described (17, 59). Only 1 shRNA (TRCN0000008415) was able to completely reduce *Hdac6* levels (59). *Erbb2* knockdown cells were created with TRCN0000023384, TRCN0000023385, and TRCN0000023386. *Fgfr4* knockdown cells were created with TRCN0000023564, TRCN0000023565, and TRCN0000023566.

### Doubling time

The mean doubling times were calculated in hours for the specified TS cells using the online computing program from Roth V. Doubling Time Computing, Available from: http://www.doubling-time.com/compute.php. Initial concentration was the number of cells set, final concentration was the number of cells counted at the end of 4 days, and duration was the period of 96 h.

### *Map3k4*^*KI*^ genotyping

Genotyping of the MAP3K4 KI mutation was performed as previously described (18). Briefly, mouse tail snips or embryonic tail snips were digested overnight in 50 mM Tris, 0.4 M NaCl, 100 mM EDTA, and 0.5% SDS and 0.58 mg/ml proteinase K at 55 °C. On the next day, the supernatant was treated with saturated NaCl and spun at room temperature. Then, 0.7 ml of the supernatant was transferred, 0.8 ml of 100% EtOH was added, and centrifuged at 4 °C. The remaining DNA pellet was then washed with 70% EtOH and resuspended in water. Genotyping for *Map3k4*^*KI*^ was performed using PCR with Platinum Taq DNA polymerase (Invitrogen). Primers for *Map3k4* and the neomycin insert in *Map3k4*^*KI*^ are listed in Table S4. PCR products were resolved in a 1% agarose gel with ethidium bromide and the Bio-Rad Chemidoc Touch for imaging. Genotyping for sex chromosomes was performed as previously described (18).

### RTK arrays

RTK array analyses (R&D Systems; catalog no.: ARY014) were performed according to the manufacturer’s instructions. Briefly, cells were harvested in RTK array lysis buffer with protease inhibitors and phosphatase inhibitors. Lysates (450 μg) were incubated overnight at 4 °C with the RTK antibody array membranes. On the next day, the arrays were washed and incubated with anti–phospho-tyrosine-horseradish peroxidase detection antibody provided by the kit for 2 h at room temperature. Arrays were washed and developed using Clarity Western ECL substrate (Bio-Rad; catalog no.: 170-5061). The RTK array blots were imaged using either film or a Bio-Rad Chemidoc Touch.

### Cell lysis and Western blotting

Whole-cell lysates of TS cells were harvested as previously described (15, 16). Briefly, whole-cell lysates were harvested in buffer A (20 mM Tris [pH 7.4], 150 mM NaCl, 1 mM EDTA, 1 mM EGTA, and 1% Triton X) with protease and phosphatase inhibitors as described previously (18). Radioimmunoprecipitation assay extractions of placentas were homogenized for a 5 s burst on ice in radioimmunoprecipitation assay buffer (buffer A, 0.1 mg/ml SDS, and 0.1 mg/ml sodium deoxycholate) with protease and phosphatase inhibitors as described for whole-cell lysates. Placental lysates were then spun at 16,200*g* for 10 min at 4 °C. Nuclear extracts were isolated as previously described (16, 17, 60). Lysates were blotted with the indicated antibodies described in Table S5. Imaging was performed using either film or the Bio-Rad Chemidoc Touch. Densitometry was measured using Bio-Rad Image Lab software.

### Real-time qPCR

RNA was isolated from TS cells using the RNeasy Plus mini kit from QIAGEN. Complementary DNA was prepared from 3 μg of RNA, using the High-Capacity reverse transcription kit from Thermo Fisher Scientific. Gene expression changes were measured using the Bio-Rad CFX96 Touch in combination with iTaq. Gene expression was normalized to *Rps11* or *Gapdh* as indicated in the figure legend. Primers are described in Table S4. Expression levels were calculated using the 2^−ΔΔCT^ method and normalized to undifferentiated TS^WT^ cells.

### Statistics and analyses

Statistical analyses were performed using Prism 9 (GraphPad Software, Inc). Embryonic and placental parameters, densitometry of Western blots, and qPCR data were analyzed using two-tailed unpaired Student’s *t* test and two-way ANOVA. Information about statistical methods for all experiments including statistical tests used and number of individuals or replicates are defined in each figure legend. ∗*p* < 0.05, ∗∗*p* < 0.01, ∗∗∗*p* < 0.001, and ∗∗∗∗*p* < 0.0001 were considered statistically significant.

## Data availability

The authors declare that all data underlying this work are present in the published article.

## Supporting information

This article contains supporting information.

## Conflict of interest

The authors declare that they have no conflicts of interest with the contents of this article.
