# Supplementary material for: MAP3K4 promotes fetal and placental growth by controlling the receptor tyrosine kinases IGF1R/IR and Akt signaling pathway
Source: J Biol Chem. 2022 Jul 31;298(9):102310. doi: 10.1016/j.jbc.2022.102310 (PMC9463538; doi:10.1016/j.jbc.2022.102310)
Supplement: Supplemental Tables S1–S5 and Figures S1–S5 [file mmc1.pdf]

MAP3K4 promotes fetal and placental growth by controlling the receptor tyrosine kinases IGF1R/IR and Akt signaling pathway†

Charles H. Perry[1‡], Nathan A. Mullins[1‡], Razan B.A. Sweileh[1], Noha A.M. Shendy[1,2], Patrick A. Roberto[1], Amber L. Broadhurst[1], Hannah A. Nelson[1], Gustavo A. Miranda-Carboni[3], and Amy N. Abell[1 *]

Table S1

Table S2

Table S3

Table S4

Table S5

Figure S1

Figure S2

Figure S3

Figure S4

Figure S5

Table S1: Reduced numbers at weaning of mice with homozygous deficiency in MAP3K4 kinase activity in the F5 C57BL/6N background.

Map3k4 Genotype	Male	Female	Total
WT/WT	19 (20.21%)	15 (15.96%)	34 (36.17%)
WT/KI	32 (34.04%)	25 (26.60%)	57 (60.64)
KI/KI	0 (0.0%)	3 (3.19%)	3 (3.19%)
Total	51 (54.3%)	43 (45.7%)	94 (100%)

*Data show mice sired over a 14-month period.
**Of the three phenotypic female *Map3k4*$^{KI/KI}$ mice, all three were XY.

Table S2. Mean weights of *Map3k4*$^{KI/KI}$ kinase-inactive mice in the 129/SvEv background.

Map3k4 Genotype	5-6 Month Female Weight (gm) ± SD	n	5-6 Month Male Weight (gm) ± SD	n	10-12 Month Female Weight (gm) ± SD	n	10-12 Month Male Weight (gm) ± SD	n
WT/WT	25.74 ± 1.98	7	31.82 ± 4.58	6	27.85 ± 1.88	4	35.96 ± 4.55	7
WT/KI	23.99 ± 0.69	5	30.95 ± 4.42	15	27.92 ± 3.36	4	33.28 ± 3.11	5
KI/KI	19.14 ± 1.86	4	25.58 ± 4.0	7	20.27 ± 2.15	3	24.89 ± 5.37	3

Table S3. Mean values ± SD of *Map3k4*$^{KI/KI}$ kinase-inactive embryo parameters at E13.5 in the 129/SvEv background.

Map3k4 Genotype	n of Embryos	Embryo Length (mm) ± SD	Liver Area (mm^2) ± SD	Placental Weight (mg) ± SD	Placental Area (mm^2) ± SD
WT/WT	8	10.01 ± 0.40	5.52 ± 0.79	66.14 ± 13.55	28.77 ± 3.54
WT/KI	15	9.88 ± 0.43	5.00 ± 0.85	63.73 ± 11.93	25.68 ± 2.32
KI/KI	9	9.54 ± 0.36	4.44 ± 1.26	50.26 ± 9.57	23.10 ± 2.82

Table S4. Primer list

Application	Primer	Sequence 5'-3'
Map3k4 Genotyping	*Map3k4* F	CTGCATCAGTGTTGACACAGGGGA
	Map3k4 R	GTGAAGCTCCACGCCAAAATACCG
	Neomycin F	CCGACTGCATCTGCGTGTTCG
	Map3k4 R2	GGGATGTGAGCGACTCTGGCCTTA
qPCR	*Rps11* F	CGCGTGGTGAATAAGGAAGC
	Rps11 R	GTAAGCACGCTCCGTCTGAA
	Cdx2 F	AAGCCAAGTGAAAACCAGGACA
	Cdx2 R	GGCAGCCAGCTCACTTTTC
	Crebbp F	GGCTTCTCCGCGAATGACAA
	Crebbp R	GTTTGGACGCAGCATCTGGA
	Erbb2 F	GAGACAGAGCTAAGGAAGCTGA
	Erbb2 R	ACGGGGATTTTCACGTTCTCC
	Esrrb F	GGACTCGCCGCCTATGTTC
	Esrrb R	CGTTAAGCATGTACTCGCATTTG
	Fgfr4 F	GCTCGGAGGTAGAGGTCTTGT
	Fgfr4 R	CCACGCTGACTGGTAGGAA
	Gapdh F	AGGTCGGTGTGAACGGATTTG
	Gapdh R	TGTAGACCATGTAGTTGAGGTCA

	Hdac6 F	TCCACCGGCCAAGATTCTTC
	Hdac6 R	CAGCACACTTCTTTCCACCAC
	Igf1r F	TCCTCCAACTCTGCCCAATG
	Igf1r R	TCTGATGGGTCTTTTGTCTTTGGA
	Insr F	CAGTGAGTGCTGCTGATGCC
	Insr R	TGCCTGAAGTTTTTCTG
	Pdpk1 F	GTG CCC ATT CAG TCC AGT GT
	Pdpk1 R	AAG GGG TTG GTG CTT GGT C
	Prl2c2 F	TCAACCATGCTCCTGGATACTG
	Prl2c2 R	GGCAACATTCTTCCACAATAACG
	Synb F	TCGTCACCACCTTCTCACTG
	Synb R	TGGCTGTAGGCTCTCAGGTT

Table S5. Antibodies and sources

Antibody	Vendor	Part #	RRID #
Akt1	Cell Signaling	2938	AB_915788
Phospho-Akt(Thr308)	Cell Signaling	13038	AB_2629447
Phospho-Akt(Ser473)	Cell Signaling	9271	AB_329825
CBP	Cell Signaling	7389	AB_2616020
Donkey anti-mouse peroxidase conjugate	Jackson ImmunoResearch Labs	715-035-151	AB_2340771
Donkey anti-rabbit peroxidase conjugate	Jackson ImmunoResearch Labs	711-035-152	AB_10015282
ERK2	Santa Cruz	sc-154	AB_2141292
Phospho-ERK1/2 (Thr202/Tyr204)	Cell Signaling	4370	AB_2315112
ERBB2	Cell Signaling	2165	AB_10692490
Phospho-ERBB2 (Tyr1248)	Cell Signaling	2247	AB_331725
FGFR4	R&D Systems	AF2265	AB_2231696
GSK3α/β	Cell Signaling	5676	AB_10547140
Phospho-GSK3α(Ser21)	Cell Signaling	9316	AB_659836
Phospho-GSK3β(Ser9)	Cell Signaling	5558	AB_10013750

HDAC6	Cell Signaling	7612	AB_10889735
IGF1Rβ	Cell Signaling	9750	AB_10950969
IRβ	Cell Signaling	3025	AB_2280448
Lamin B1	Cell Signaling	13435	AB_2737428
Phospho-IGF1Rβ(Tyr1131)/IRβ(Tyr1146)	Cell Signaling	3021	AB_331578
Phospho-IGF1Rβ (Tyr1135/1136)/IRβ(Tyr1150/1151)	Cell Signaling	3024	AB_331253
Phospho-JNK (Thr183/Tyr185)	Cell Signaling	9251	AB_331659
Phospho-p38 (Thr180/Try182)	Cell Signaling	9211	AB_331641
PDK1	Cell Signaling	5662	AB_10839264
Phospho-PDK1(Ser241)	Cell Signaling	3438	AB_2161134
PTEN	Cell Signaling	9188	AB_2253290
Phospho-PTEN (Ser380/Thr382/Thr383)	Cell Signaling	9549	AB_659891
α-tubulin	Sigma-Aldrich	T9026	AB_477593

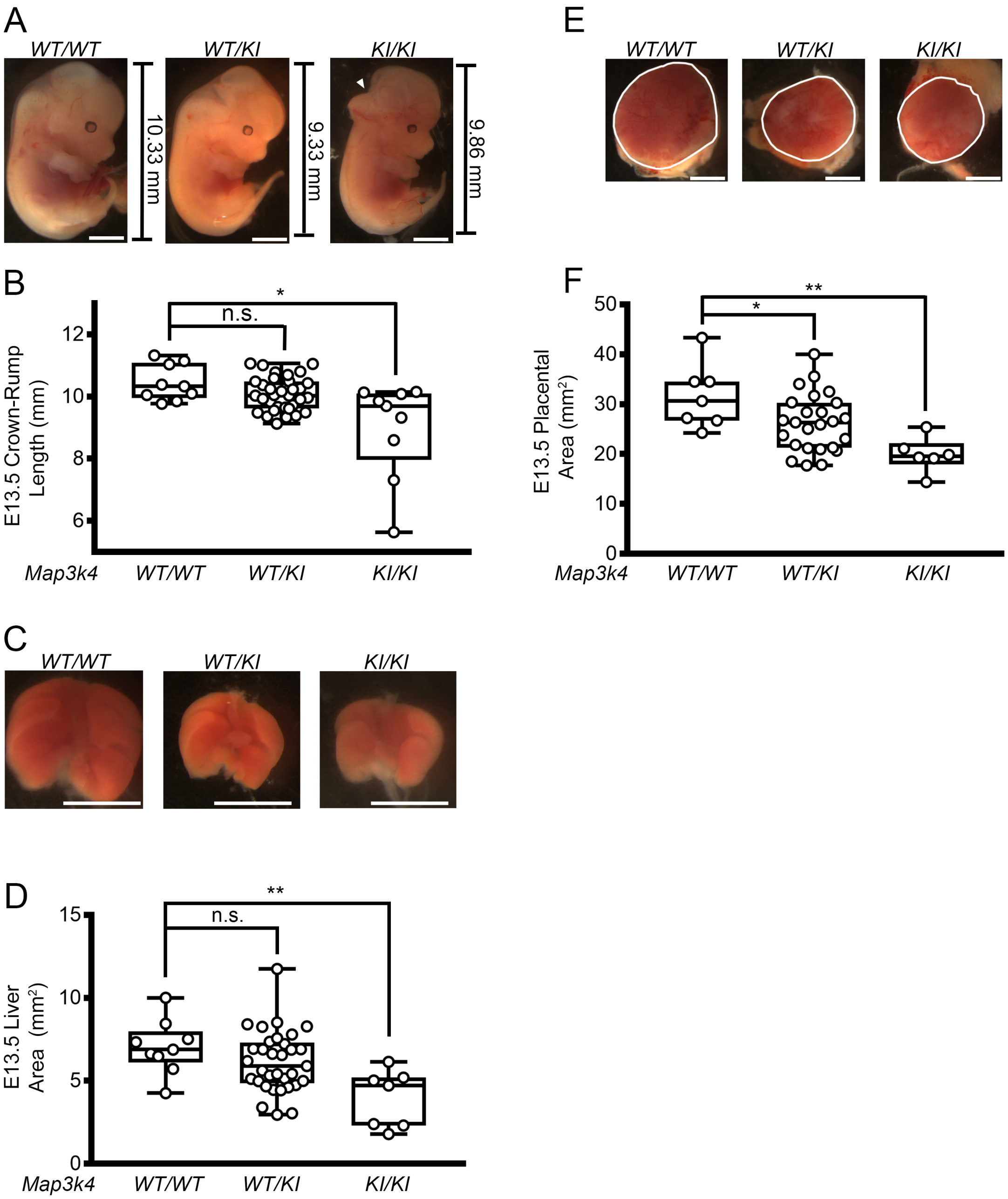

Figure S1. Reduced embryo length, liver area, and placental area in E13.5 *Map3k4*$^{KI/KI}$ embryos in a mixed 129/SvEv/C57BL/6N background.

A, representative images of E13.5 *Map3k4*$^{WT/WT}$, *Map3k4*$^{WT/KI}$, and *Map3k4*$^{KI/KI}$ mixed background embryos. Black scale bars and numbers indicate crown-rump length. White

scale bar, 2 mm. White arrowhead indicates exencephaly. *B,* reduced crown-rump length of *Map3k4*$^{KI/KI}$ E13.5 embryos compared to *Map3k4*$^{WT/WT}$ E13.5 embryos. *C*, representative images of *Map3k4*$^{WT/WT}$, *Map3k4*$^{WT/KI}$, and *Map3k4*$^{KI/KI}$ E13.5 livers. White scale bar, 2 mm. *D*, reduced liver area of *Map3k4*$^{KI/KI}$ embryos compared to *Map3k4*$^{WT/WT}$ livers. *E***,** representative images of E13.5 *Map3k4*$^{WT/WT}$, *Map3k4*$^{WT/KI}$, and *Map3k4*$^{KI/KI}$ placentas outlined in white. White scale bar, 2 mm. *F*, reduced placental area of *Map3k4*$^{WT/KI}$ and *Map3k4*$^{KI/KI}$ placentas compared to *Map3k4*$^{WT/WT}$ placentas. *B, D*, and *F,* data are displayed as box plots; each dot represents one individual. * *p*-value < 0.05; ** *p*-value < 0.01; Student's *t* test; n.s., not significant.

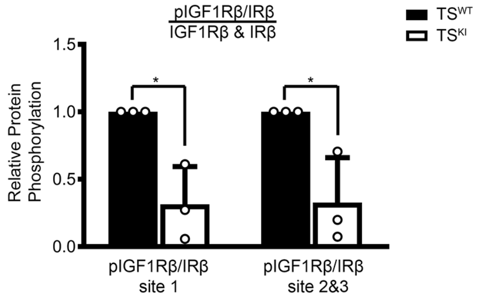

Figure S2. Densitometry of Western blots detecting basal phosphorylated IGF1Rβ and IRβ normalized to combined total IGF1Rβ and IRβ.

Densitometry analyses of basal phosphorylation of IGF1Rβ/IRβ normalized to combined total IGF1Rβ and IRβ. Graphs show the mean ± SD of three biologically independent experiments. * *p*-value < 0.05; Student's *t* test.

A

Treatment	Mean Doubling Time (Hours)	Passages	SD	p-value
TS^KI	15.92	3	0.231	N/A
TS^KI Fgfr4sh1	21.02	3	1.034	0.001
TS^KI Fgfr4sh2	17.79	3	1.48	0.144
TS^KI Fgfr4sh3	16.18	3	0.083	0.14

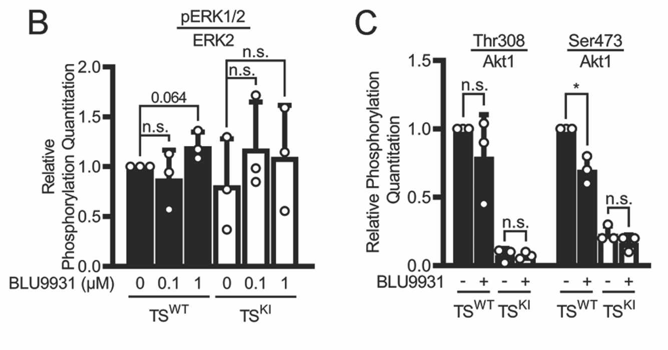

Figure S3. Cellular doubling time with shRNA knockdown of *Fgfr4* in TS^{KI} cells.

A, Cellular doubling time was measured over 96 hours in control infected TS^{KI} cells or cells stably expressing three independent *Fgfr4* shRNAs. *B,* densitometry analyses of Western blots from TS^{WT} or TS^{KI} cells treated for 48 hours with either vehicle control (DMSO), or the indicated concentration of BLU9931. Densitometry of phosphorylated ERK1/2 was normalized to total ERK2 expression. Plots show the mean ± SD of three biologically independent experiments. *C,* densitometry analyses of Western blots of TS^{WT} or TS^{KI} cells treated for one hour with either vehicle control (DMSO) or 1 μM BLU9931. Densitometry of phosphorylated Akt at either Thr308 or Ser473 was normalized to total Akt1 expression. Plots show the mean ± SD of three biologically independent experiments. * *p*-value < 0.05; Student's *t* test; n.s., not significant.

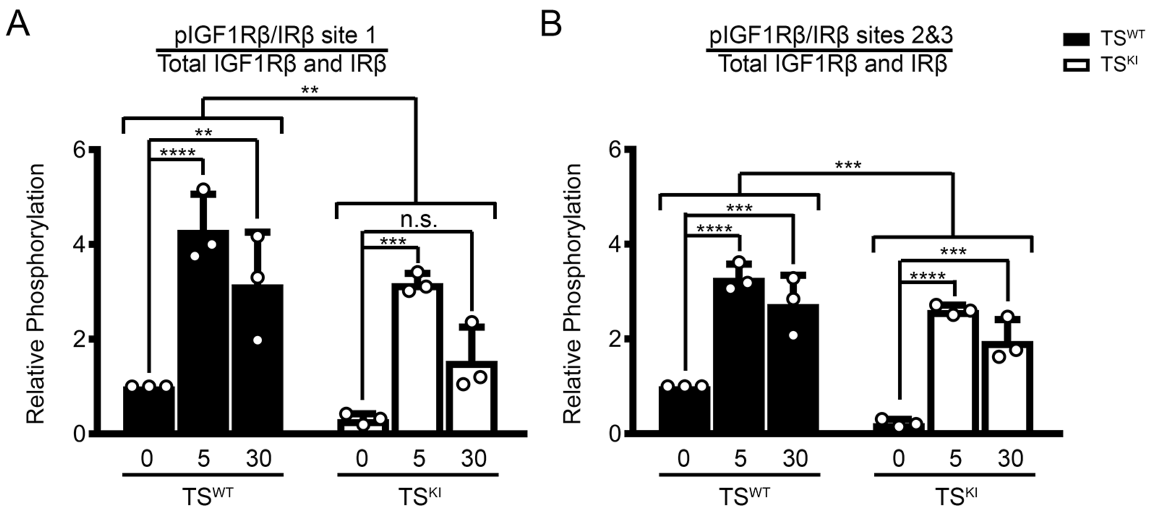

Figure S4. Densitometry of Western blots detecting insulin-stimulated phosphorylated IGF1Rβ and IRβ normalized to combined total IGF1Rβ and IRβ. *A,B*, densitometry analyses of insulin-stimulated phosphorylation of IGF1Rβ/IRβ normalized to combined total IGF1Rβ and IRβ. Site 1 represents pIGF1Rβ Tyr1131 and pIRβ Tyr1146, site 2 represents IGF1Rβ Tyr1135 and pIRβ Tyr1150, and site 3 represents pIGF1Rβ Tyr1136 and pIRβ Tyr1151. Densitometry was used to quantify three biologically independent experiments. Graphs show the mean ± SD. ** *p*-value < 0.01; *** *p*-value < 0.001; **** p-value < 0.0001; Student's *t* test; two-way ANOVA; n.s., not significant.

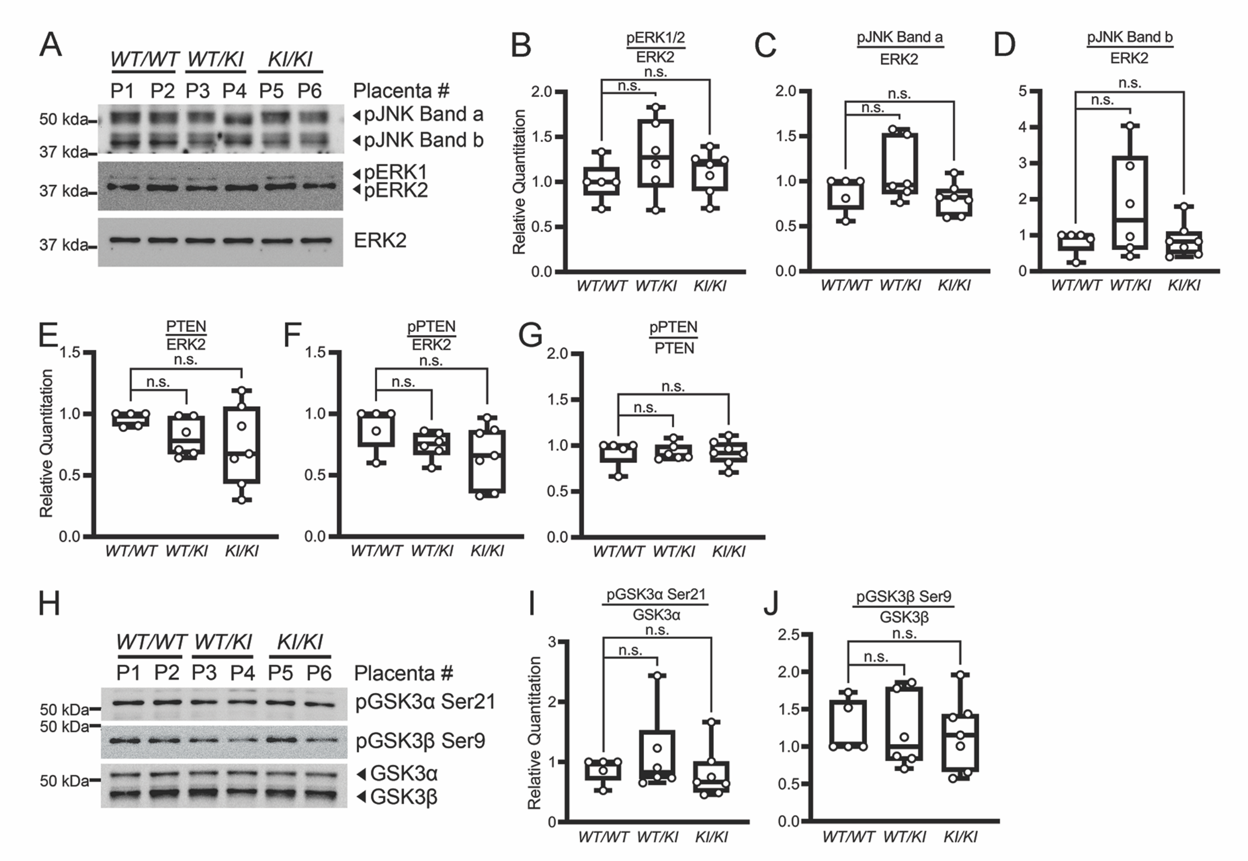

Figure S5. Protein expression and phosphorylation in E13.5 placentas.

A-J, Expression and phosphorylation were measured in eighteen independent placental extractions. *A,* ERK and JNK phosphorylation are not altered in *Map3k4*$^{KI/KI}$ E13.5 placentas. Phosphorylated JNK bands are labeled bands a and b. *B-D,* densitometry analyses are shown with phosphorylation normalized to ERK2. *E-G,* densitometry analyses are shown with *(E)* PTEN protein or *(F)* phosphorylated PTEN normalized to ERK2 protein, or *(G)* phosphorylated PTEN normalized to PTEN protein. *H-J,* GSK3α and GSK3β phosphorylation is not significantly altered in *Map3k4*$^{KI/KI}$ E13.5 placentas as measured by Western blotting. *I, J,* densitometry analyses are shown. Phosphorylated GSK3α was normalized to total GSK3α protein, and phosphorylated

GSK3β was normalized to total GSK3β protein. *A* and *H*, Western blot images show two independent placentas for each genotype and are representative of eighteen independent placental extractions. *B*-*G* and *I*-*J,* densitometry analyses of Western blots were used to quantify eighteen independent placental extractions. Data are displayed as box plots; each point represents one individual placenta. Student's *t* test; n.s., not significant.
